# Revisiting the Electrochemical Nitrogen Reduction on Molybdenum and Iron Carbides: Promising Catalysts or False Positives?

**DOI:** 10.1021/acscatal.2c04491

**Published:** 2023-01-12

**Authors:** Boaz Izelaar, Davide Ripepi, Simone Asperti, A. Iulian Dugulan, Ruud W.A. Hendrikx, Amarante J. Böttger, Fokko M. Mulder, Ruud Kortlever

**Affiliations:** † Large Scale Energy Storage, Process and Energy Department, Faculty of Mechanical, Maritime and Materials Engineering, Delft University of Technology, Delft2628 CB, The Netherlands; ‡ Materials for Energy Conversion and Storage, Chemical Engineering Department, Faculty of Applied Sciences, Delft University of Technology, Delft2629 HZ, The Netherlands; § Radiation Science and Technology Department, Faculty of Applied Sciences, Delft University of Technology, Delft2629 HZ, The Netherlands; ∥ Surface and Interface Engineering, Materials Science and Engineering Department, Faculty of Mechanical, Maritime and Materials Engineering, Delft University of Technology, Delft2628 CB, The Netherlands

**Keywords:** electrocatalysis, nitrogen reduction reaction, ammonia, molybdenum, iron, carbide, impurities

## Abstract

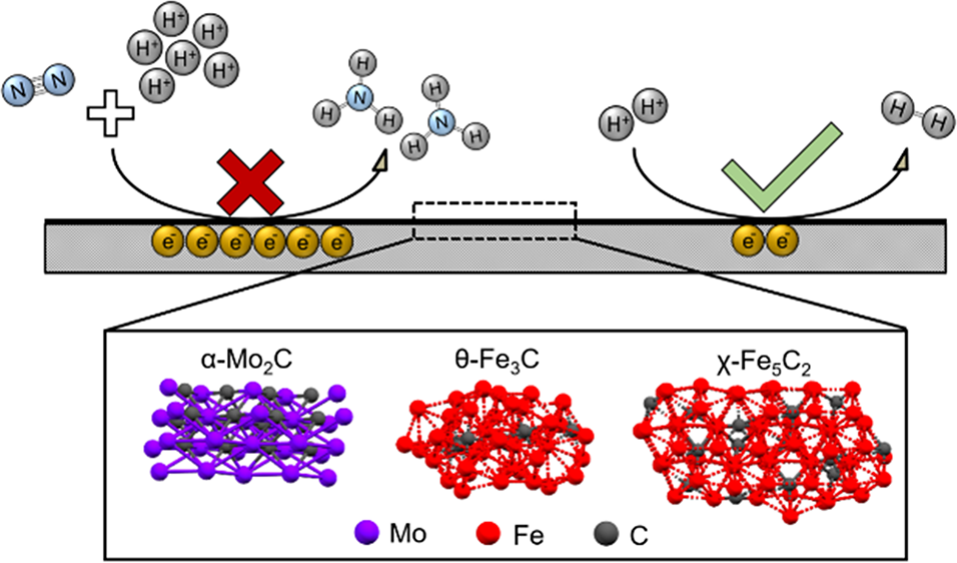

The electrochemical dinitrogen reduction reaction (NRR) has recently gained much interest as it can potentially produce ammonia from renewable intermittent electricity and replace the Haber–Bosch process. Previous literature studies report Fe- and Mo-carbides as promising electrocatalysts for the NRR with activities higher than other metals. However, recent understanding of extraneous ammonia and nitrogen oxide contaminations have challenged previously published results. Here, we critically assess the NRR performance of several Fe- and Mo-carbides reported as promising by implementing a strict experimental protocol to minimize the effect of impurities. The successful synthesis of α-Mo_2_C decorated carbon nanosheets, α-Mo_2_C nanoparticles, θ-Fe_3_C nanoparticles, and χ-Fe_5_C_2_ nanoparticles was confirmed by X-ray diffraction, scanning and transmission electron microscopy, and X-ray photoelectron and Mössbauer spectroscopy. After performing NRR chronoamperometric tests with the synthesized materials, the ammonia concentrations varied between 37 and 124 ppb and are in close proximity with the estimated ammonia background level. Notwithstanding the impracticality of these extremely low ammonia yields, the observed ammonia did not originate from the electrochemical nitrogen reduction but from unavoidable extraneous ammonia and NO_*x*_ impurities. These findings are in contradiction with earlier literature studies and show that these carbide materials are not active for the NRR under the employed conditions. This further emphasizes the importance of a strict protocol in order to distinguish between a promising NRR catalyst and a false positive.

## 1 Introduction

The activation of diatomic nitrogen has been one of the greatest challenges in nitrogen-related chemistry.^1,2^ This is inherently related to the inert nature of the nitrogen molecule due to its high bond-dissociation energy, absence of a dipole moment, and low proton and electron affinity.^3^ Despite the inert nature, diazotrophic microorganisms successfully fixate nitrogen and play a key role in enriching the soil.^4^ However, due to the growing world population and the high demand for food, additional nitrogen containing nutrients in the form of artificial ammonia-based fertilizers must be provided to the soil. The majority of the ammonia produced worldwide is synthesized by the Haber–Bosch process (H-B), wherein energy intensive reaction conditions (*T* = 300–500 °C, *P* = 150–300 bar) are needed to activate dinitrogen.^2^ The ammonia industry consumes approximately 1% of the global energy demand and emits roughly 0.75% of the anthropogenic CO_2_ emissions (assuming 1.9 t_CO_2__ t_NH_3__^–1^),^5,6^ which is motivating the search for more energy efficient and sustainable alternatives.

The electrochemical nitrogen reduction reaction (NRR), wherein dinitrogen, water, and electrons from renewable sources react to form ammonia, has recently gained significant scientific interest and has been proposed as a potential replacement for the fossil fuel-based H-B.^7,8^ NRR systems at high (>500 °C) and intermediate (100–500 °C) temperatures have proven to be successful in terms of faradaic efficiency (FE ≥ 75%) and NH_3_ yield (≥4.5 nmol s^–1^ cm^–2^).^9^ Nevertheless, the present high temperature NRR systems tend to have a low energy efficiency compared to H-B.^10,11^ Therefore, it would be beneficial to perform the NRR under ambient conditions. Significant FEs have been reported with iron electrocatalysts in ionic liquids and organic electrolytes by suppressing the parasitic hydrogen evolution reaction (HER).^12,13^ However, the use of organic electrolytes is likely to be less economically feasible compared to aqueous electrolytes due to complex scalability, safety issues, high costs, and intense energy requirements.^8,14^ Unfortunately, the kinetics under ambient conditions in aqueous electrolytes are sluggish and many attempts in examining transition metals, such as Au, Fe, Mo, Ru, Rh, and Re, as potential electrocatalysts for the NRR have resulted in low FEs (<1%) and ammonia yields (<0.1 nmol s^–1^ cm^–2^).^15,16^

The active site of the nitrogenase enzyme, the biologic pathway for nitrogen fixation, is the FeMo-cofactor. The FeMo-cofactor contains a six iron atomic trigonal prism with a carbon-centered position. Each iron is bound to three sulfur atoms, with an additional iron and molybdenum in apical positions.^2,17^ Attempts to mimic the FeMo-cofactor initiated investigation into Fe- and Mo-based heterogeneous NRR catalysts, such as carbides and sulfides. Both Mo_2_C and MoS_2_ show noble metal like properties, due to similar d-band configurations as Pt.^18^ Therefore, they can act as cheap and robust catalytic substitutes for many applications, including water electrolysis, water gas shift reaction, and ammonia decomposition.^19^ Despite the fact that these materials are good HER catalysts, several density functional theory studies have predicted favorable nitrogen binding energies.^19−21^ Experimental results are somewhat distinct; nitrogen reduction experiments with amorphous 2H-MoS_2_ and metallic 1 T-MoS_2_ did not produce quantities of ammonia exceeding the background level,^12,21^ while other studies report reasonable FEs and ammonia yields using FeS_2_, Mo_2_C, and Fe_3_C, thereby labeling these materials as promising NRR catalysts.^22−24^

The electrochemical NRR field is plagued by questionable results, mainly due to the large impact of extraneous ammonia sources on experiments performed on a small scale. Ammonia stemming for other sources can erroneously be assigned to ammonia synthesized by the NRR, which can lead to false positives. Ammonia impurities can be minimized by a proper experiment design and can be identified by applying the right control experiments, such as argon and open-circuit blank tests and ultimately ^15^N_2_-labeled experiments. Recently, nitrogen oxides have been identified as another source of contamination, as these species are more easily reduced to ammonia than dinitrogen in the NRR.^25^ The majority of the recently published studies have applied blank tests, but performing quantitative ^15^N_2_-labeled experiments and monitoring nitrogen oxide species are done sporadically.^25^ As a consequence, a handful of research groups have tried to reproduce electrocatalysts initially labeled as promising, such as Fe, Bi, Au, VN, CoMo, Mo_2_N, and MoS_2_,^16,21,26−30^ but discovered that the quantified ammonia must originate from sources other than the NRR. Here, we critically assess the electrocatalytic NRR activity of molybdenum and iron carbides, where more than 10 independent literature reports claim to observe superior or excellent catalytic performance.^23,24,31−38^ In the present work, α-Mo_2_C nanodots from Cheng et al. (reported as the most promising carbide catalyst) are reproduced and compared with α-Mo_2_C nanoparticles as a benchmark.^23^ Additionally, nanostructured θ-Fe_3_C and χ-Fe_5_C_2_ are synthesized and examined for their NRR activity. A key aspect of this work is the implementation of a strict protocol, which is designed to minimize the level of extraneous contamination,^15,25^ allowing genuine quantification of ammonia produced by the NRR.

## 2 Experimental Section

### 2.1 Materials

Materials and chemicals were purchased from Sigma Aldrich, if not indicated otherwise. Ultrapure water (Millipore Milli-Q IQ 7000) was used for catalyst synthesis, electrolyte preparation, and cleaning procedures. Concentrated sulfuric acid (95–98 wt % H_2_SO_4_, trace metal purity) was used for glassware acid cleaning and diluted for other purposes. High purity N_2_, Ar, and H_2_ (99.999%, Linde) were used for electrochemical experiments and material synthesis.

### 2.2 Molybdenum and Iron Carbide Synthesis

#### 2.2.1 Mo_2_C Nanodot-Decorated Carbon Nanosheets

Mo_2_C nanodots (Mo_2_C NS) were synthesized by a molten-salt synthesis procedure as reported in detail elsewhere.^23^ In short, a mixture of 1 mL of water and 4 mL of ethanol (96%, VWR) was mixed in a beaker and continuously heated and stirred on a hotplate. Once the mixture reached 70 °C, 0.4 g of bis(acetylacetonato)dioxomolybdenum(VI) and 0.14 g of sucrose (99.5%) were added. After the mixture turned green, an excess amount of sodium chloride (99.5%) was added until a green crystalline slurry was formed. The slurry was directly transferred to a ceramic boat and placed inside a tubular furnace (Blue, Lenton), where the specimen was heated to its carburization temperature under an Ar atmosphere (*T*_carb_ = 800 °C, heating rate = 5 °C min^–1^), kept constant at this temperature for 2 h and the furnace cooled down to room temperature naturally. The resulting black catalyst/salt mixture was excessively rinsed with ultrapure water to remove the sodium chloride. The residue was filtrated (Durapore 100 nm, Merck) and dried in an oven at 80 °C overnight.

#### 2.2.2 α-Mo_2_C Nanoparticles

Gómez-Marín and Ticianelli reported a procedure for the synthesis of porous Mo_2_C nanoparticles (Mo_2_C NP) that was replicated here.^39^ In a typical procedure, 0.15 g of Vulcan VC-72 (Cabot) was mixed with 0.51 g of MoO_3_ (99.9%) in a beaker containing 30 mL of ethanol. The dispersion was heated to 60 °C overnight while continuously stirring to evaporate the ethanol completely. The powder was transferred to a ceramic boat for carburization inside a tubular furnace under 10 vol % H_2_:Ar. The precursor was heated to 725 °C for 30 min with a slow heating rate (1 °C min^–1^) and cooled down to room temperature.

#### 2.2.3 Mesoporous Fe_3_C

A combined hard-templating and carburization method developed by Kraupner and coworkers was used to create a mesoporous Fe_3_C structure with a high surface area.^40^ In brief, 0.5 g of FeCl_3_ (99.9%) was dissolved in 1 g of 40 wt % SiO_2_ in H_2_O (Ludox AS40) in a borosilicate test tube. Additionally, 0.728 g of 4.5-dicyanoimidazole (99%) was added and stirred through the suspension and sonicated for 30 min to achieve a homogeneous yellow-colored thick slurry paste. The paste was transferred to a ceramic boat and carburized inside a tubular furnace at 700 °C (heating rate 2 °C min^–1^) for 2 h under an Ar atmosphere and cooled down to room temperature.

#### 2.2.4 χ-Fe_5_C_2_ Nanoparticles

The principle of thermo-decomposition of Fe(CO)_5_ is a common used strategy to synthesize iron carbides and is discussed in detail elsewhere.^41^ A mixture of 0.2 g of polyvinylpiyrrolidone (PVP, 40000 g/mol) and 1 mL of Fe(CO)_5_ (99.99%) was inserted in a homemade air-tight reactor consisting of Swagelock tubes and adapters (Figure S1). The reactor was purged with Ar at a flowrate of 20 mL min^–1^ for approximately 10 min to remove residual oxygen, and immediately afterward, all Swagelock adapters were closed. The reactor was positioned inside a muffle furnace programmed with *T*_carb_ at 300 °C (heating rate 2.3 °C min^–1^) for a duration of 24 h.

### 2.3 Characterization

#### 2.3.1 X-ray Diffraction (XRD)

Samples were deposited on a Si510 zero background wafer and positioned inside a Bruker D8 Advance diffractometer in Bragg–Brentano geometry equipped with a graphite monochromator, a Vantec position sensitive detector, a variable divergence slit, and a 5 mm height scatter screen. Co Kα radiation (λ = 0.1789 nm) was used to avoid incident beam fluorescence effects on the Fe carbides. During each acquisition, steps with a size of 0.038° and 5 s per step were measured over a 10–110° 2θ range. Bruker DiffracSuite.EVA v6.0 was used to subtract the background, correct small displacements, and strip the Kα2 contribution from the patterns to enable crystallite size (*D*_XRD_) estimation with the Scherrer equation (eq 1), where λ is the wavelength, and κ the shape factor taken as 1. Peak shapes were assumed Gaussian, and the full width at half-maximum, in this case β, was additionally corrected for instrumental line broadening effects.
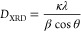
1

#### 2.3.2 Mössbauer Spectroscopy

Transmission ^57^Fe Mössbauer spectra were collected at room temperature with a conventional constant-acceleration spectrometer with a ^57^Co(Rh) source. Velocity calibration was carried out using an α-Fe foil. The Mössbauer spectra were fitted using the Mosswinn 4.0 program.^42^

#### 2.3.3 X-ray Photoelectron Spectroscopy (XPS)

A Thermo Scientific Kα spectrometer with a monochromatic Al Kα excitation source was used to acquire X-ray photoemission spectroscopy (XPS) spectra. The base pressure inside the analysis chamber was about 2 × 10^9^ mbar. HR-XPS spectra were recorded using a 400 μm spot size, 0.1 eV step size, and 50 eV pass energy (200 eV for survey). All spectra were charge-corrected to the C 1s adventitious carbon (284.8 eV). Subsurface layers were measured with a depth profile by argon ion etching (1000 eV) in between XPS measurements. The obtained XPS spectra were deconvoluted with CasaXPS v2.3 software.

#### 2.3.4 Inductively Coupled Plasma Atomic Emission Spectroscopy (ICP-OES)

The materials were dispersed in 35 vol % HNO_3_ overnight to dissolve the carbides. The samples were further diluted with 3 vol % HNO_3_ with an amount depending on the expected metal content. All ICP-OES measurements were performed on a SPECTRO ARCOS measured against an external calibration, with a typical detection limit of 10 ppb.

#### 2.3.5 Scanning Electron Microscopy (SEM)

Prior to analysis, the aluminum cylindrical sample holder was washed in isopropanol in an ultrasonic bath for approximately 2 min. An isopropanol based catalyst ink was drop-casted on the sample holder and positioned in a 25 mm working distance. The SEM measurements were executed on a Jeol JSM 6500F instrument at an acceleration voltage of 15 kV, coupled with an energy dispersed X-ray analysis detector (Ultradry, Thermo Scientific).

#### 2.3.6 Transmission Electron Microscopy (TEM)

A dispersion of catalyst and isopropanol was drop-casted on a TEM grid with a holey carbon film on a copper 400 mesh (EM-resolutions). All materials were analyzed with a JEOL JEM1400plus TEM at a 120 kV acceleration voltage using a single-tilt specimen holder. The TEM was equipped with a TVIPS TemCam-F416R high-resolution camera based on a custom designed CMOS architecture. ImageJ was used to estimate the particle size distribution.

### 2.4 Electrochemical Measurements

A Biologic VSP-300 potentiostat in combination with EC-Lab software was used for all electrochemical measurements. The uncompensated resistance (*R*_u_) of the system (the resistance between the reference electrode (RE) and working electrode (WE)) was measured before each cyclic voltammetry (CV) and chronoamperometry (CA) measurement. *R*_u_ was determined with potentiostatic electrochemical impedance spectroscopy at open-circuit potential, with a frequency range between 200 kHz and 0.1 Hz. The distance between the origin and the first line intersection on the *Z*_Real_-axes within the Nyquist plot represents *R*_u_ and was extracted by manual data fitting. Subsequently, the EC-Lab build-in IR compensation allowed 85% *R*_u_ compensation without adding to much distortion to the CV and CA results. Only for the CA experiments, the other 15% R_u_ was compensated after the measurement by using eq 2.
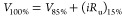
2

A polyether ether ketone (PEEK) three-electrode cell design adapted from the Jaramillo group was used for all electrochemical experiments.^43^ It consisted of two separate compartments that accommodate 5 mL of electrolyte and 3 mL of gas headspace. An additional plate was added to the overall cell design (Figure S2), which fixated the WE. A leak-free Ag/AgCl micro reference electrode (Innovative Instruments, LF-1-45) was used for potential control, wherein all potentials were recalculated versus the reversible hydrogen electrode scale following eq 3.
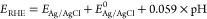
3

A Pt foil (50 × 50 × 0.025 mm, 99.99%, Mateck) functioned as the anode and was rinsed with water and flame annealed before each experiment. A fresh sheet of membrane (Celgard 3401) was used for every run, thereby preventing accumulation of NH_3_. The WE was prepared by drop-casting 3 droplets of 10 μL of a freshly prepared catalyst ink (2 mg_cat_ ml^–1^, 950 μL 2-propanol (98%, VWR) and 50 μL of Nafion 117-containing solution (5 wt %)) on a carbon paper disk (1 cm^2^, Toray carbon paper, Aesar) with a loading of 0.06 mg cm^–2^ and stored under vacuum once prepared. The WE was soaked in a fresh 1 M KOH (99.95%), 0.1 M KOH, 0.5 M Li_2_SO_4_, or 0.05 M H_2_SO_4_ solution before it was fixated in the cell by a glassy carbon plate (25 × 25 × 1 mm, HTW). The back of the glassy carbon was taped with a Cu strip (AT528, 10 mm width, RS Components) and connected to the potentiostat wires. The catholyte was saturated by purging N_2_ or Ar for 30 min before each experiment. After cyclic voltammetry and chronoamperometric measurements, aliquots of both catholyte and anolyte were collected with a syringe and transferred to several test tubes for further quantification.

### 2.5 Minimizing Effects of Impurities

Feed gas contamination in the form of NH_3_ and NO_*x*_ in both high purity Ar and N_2_ have been reported previously.^15,25^ In order to remove residual contaminants, a certified commercial gas filter (Entegris GPUS35FHX) was installed upstream of the electrochemical cell (see Figure S3). The cell components were always acid cleaned with 10 vol % H_2_SO_4_ for at least 1 h and rinsed with ultrapure water prior to each experiment. Syringes, needles, pipet tips, and sample tubes were also excessively washed with ultrapure water and dried under Ar flow before use. A microporous membrane (Celgard 3401) with a gas repellent coating was selected as a more suitable separator compared to the more commonly used Nafion membrane to avoid accumulation of ammonia contaminations as was reported previously.^15,^^44,45^ A downstream acidified liquid trap is often used to measure volatile ammonia that could potentially be present in the effluent gas. As NH_3_ dissolves very well in aqueous electrolytes (∼500 g/L), this suggests that low concentrations of NH_3_ readily dissolves in the used electrolyte. This means that an acid trap is often redundant and can potentially be an extra source of contamination.^15^ Therefore, we did not incorporate a downstream acidified trap in the experimental design.

Precursors and catalysts containing nitrogen species are potential sources of impurities and should be avoided.^28,30^ The selection criteria for our catalyst synthesis procedures was mainly motivated by minimizing the use of N-containing precursors. The Mo_2_C nanoparticles and Mo_2_C nanodots do not contain N-based materials for the preparation, while the use of N–C compounds was unavoidable for the synthesis of iron carbide nanomaterials. The latter motivated us to use a catalyst loading of 0.06 mg·cm^–2^ to minimize the effects of the N–C precursor during the electrochemical experiments. We used a method adopted from Chen et al. to monitor impurities in our materials,^26^ such as catalyst powders, membranes, carbon paper, and Pt foil. Strategies to effectively remove impurities will be discussed in a future study.^46^

Li-salts are notorious for containing trace levels of NO_*x*_^–^ species as was previously reported by Li et al.^47^ Therefore, Li_2_SO_4_ is suspected of having these labile N-species and the suggested thermal annealing step was implemented to remove trace impurities. For the annealing step, the as received Li_2_SO_4_ (99.5%) was transferred to a tubular furnace and thermally annealed at 800 °C for 4 h in Ar with a heating rate of 10 °C·min^–1^ before preparing a solution.

### 2.6 Ammonia and Nitrite Quantification

Ammonia was quantified by the Berthelot reaction.^48^ In a routine analysis, a volume of 1.33 mL of either 1 M KOH, 0.1 M KOH, 0.5 M Li_2_SO_4_, or 0.05 M H_2_SO_4_ was neutralized with dilute concentrations of H_2_SO_4_ or KOH. Then, phenol nitroprusside and alkaline hypochlorite (0.2 wt % sodium hypochlorite in an alkaline solution) were both added in an amount equal to 25 vol % of the neutralized solution. The mixture was stirred thoroughly on a vortex shaker. After 30 min of incubation time, the solution color and its intensity differed from light green to dark blue with increasing NH_3_ content. The samples were transferred to PMMA cuvettes (10 × 10 × 30 mm) for further analysis with the UV–Vis spectrophotometer (Hach DR6000). For constructing a calibration line, a series of six different concentrations of NH_4_Cl (99.99%) in 1 M KOH, 0.1 M KOH, 0.5 M Li_2_SO_4_, and 0.05 M H_2_SO_4_ were prepared with respective concentrations of 0.01, 0.05, 0.1, 0.5, 1, and 2 ppm. The fitted calibration lines shown in Figure S4 were reproducible and resulted in the following linear relationships: *A*_1MKOH_ = 0.5642C_NH_3__ – 0.0045 with *R*^2^ = 0.9997, *A*_0.1MKOH_ = 0.7279C_NH_3__ – 0.001 with *R*^2^ = 0.9999, *A*_0.5MLi_2_SO_4__ = 0.7992C_NH_3__ – 0.0033 with *R*^2^ = 0.9997, *A*_0.05MH_2_SO_4__ = 0.6613C_NH_3__ – 0.00405 with *R*^2^ = 0.9997.

The concentration of NO_2_^–^ was quantified by the photometric Griess test. A commercially available Griess reagent mixture was used with a detection range between 0.007 and 3.28 ppm NO_2_^–^ (Spectroquant, Merck). Typically, a sample of 2 mL of 0.1 M KOH was neutralized with 168 μL of 0.5 M H_2_SO_4_. Subsequently, 30 mg of the Griess reagents were added and mixed with the solution with an incubation time of 10 min. Five different concentrations of 0.02, 0.05, 0.1, 0.5, 1 ppm KNO_2_ in 0.1 M KOH were prepared to construct a calibration line with a perfect linear fit: *A* = 0.8071C_NO_2_^–^_ – 0.0001 and *R*^2^ = 1 (Figure S5). The UV–Vis spectroscopic measurements to detect ammonia and NO_2_^–^ were always performed versus a blank 0.1 M KOH electrolyte stock solution to exclude the influence of electrolyte background contaminations.

## 3 Results and Discussion

### 3.1 Material Characterization

**Figure 1:**
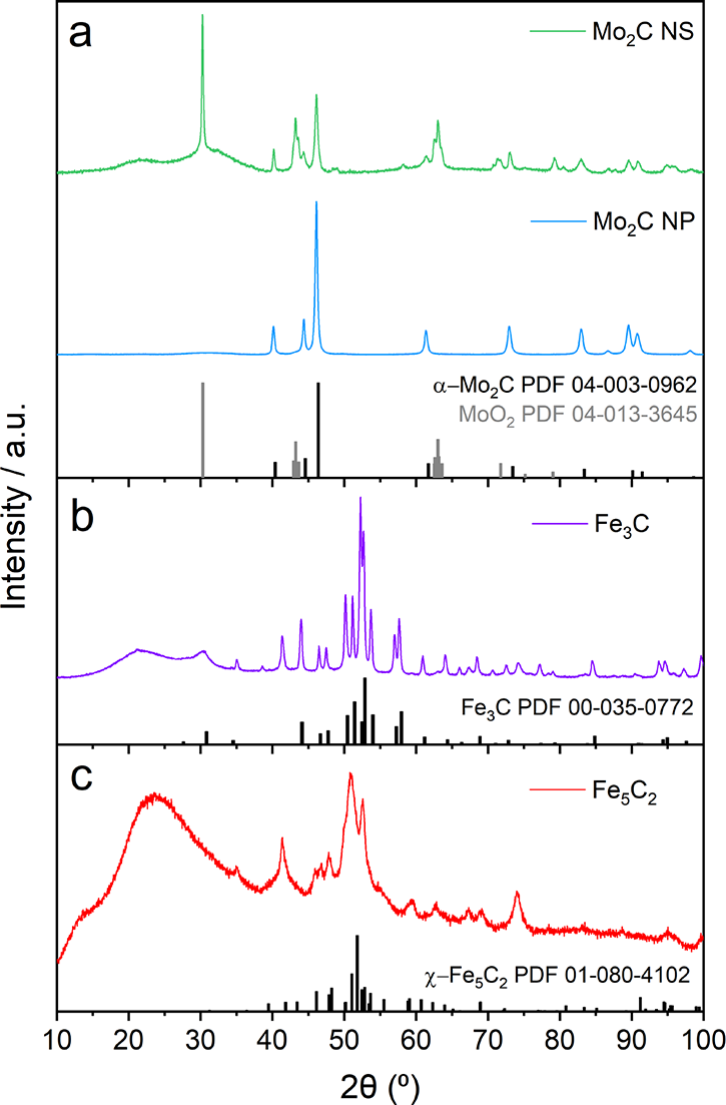
X-ray diffractions patterns of (a) Mo_2_C NS (green) and Mo_2_C NP (blue) with the α-Mo_2_C (PDF 04-003-0962, black) and MoO_2_ (PDF 04-013-3645, gray) reference patterns. The patterns in (b) and (c) represent Fe_3_C (purple) and Fe_5_C_2_ (red) with the corresponding θ-Fe_3_C (PDF 00-035-0772, black) and χ-Fe_5_C_2_ (PDF 01-080-4102, black) reference patterns.

The X-ray diffraction patterns of Mo_2_C NS, Mo_2_C NP, Fe_3_C and Fe_5_C_2_ are shown in Figure 1. The Mo_2_C samples (Figure 1a) show three sharp peaks at 40.2, 44.3, and 46.1° that are identical to the reference spectrum of α-Mo_2_C (PDF 04-003-0962). Three other peak features at 30.3, 43.2, and 63.0° suggest the existence of MoO_2_ (PDF 04-013-3645) in the Mo_2_C NS sample. This is most likely related to an incomplete carbothermal reduction of the molybdenum oxide precursor, which was not observed for the Mo_2_C NP. The ″hill-like″ peak between 20 and 25° is typical for amorphous carbon and reflects its dominant presence in the Mo_2_C NS, Fe_3_C, and Fe_5_C_2_ samples.^49^ The peaks between 45 and 60° in Figure 1b correspond to orthorhombic iron carbide (θ-Fe_3_C, PDF 00-035-0772). The formation of other Fe oxidation states, such as reduced Fe (53.3°), Fe_3_O_4_ (41.3, 35, and 74.2°), and Fe_2_O_3_ (38.6°) are inevitable by-products of the carburization process.^41,50^ Also, small fractions of Fe_3_O_4_ (41.4° and 74°) were identified in Figure 1c after the thermal decomposition of Fe(CO)_5_,^41^ while the multiplet between 49 and 55° is very typical for χ-Fe_5_C_2_ (PDF 01-080-4102). The average crystallite size was calculated with the Scherrer equation (eq 1) and summarized in Table S1.

**Figure 2:**
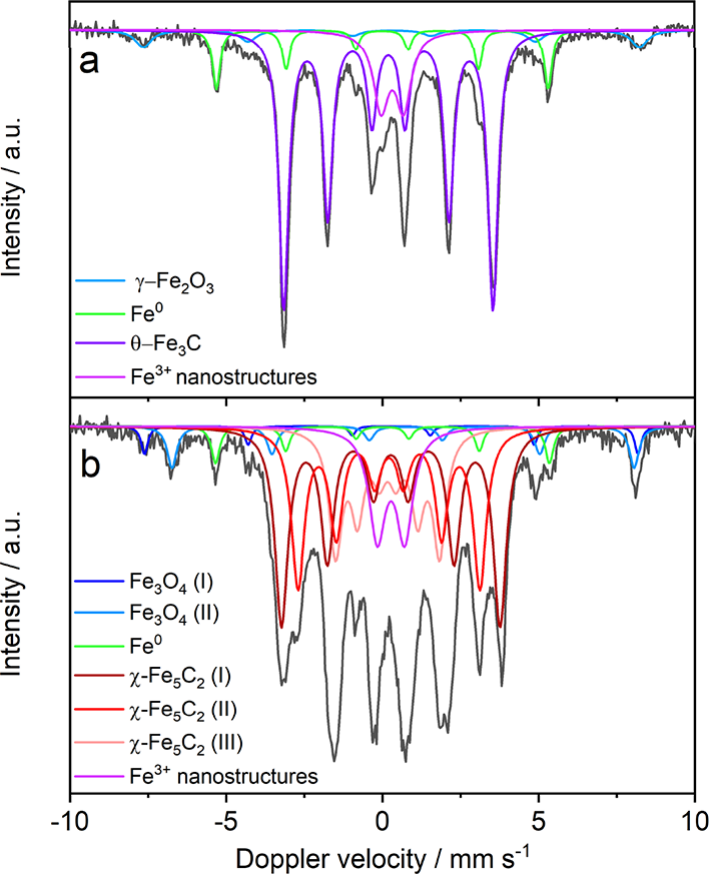
Room temperature transmission ^57^Fe Mössbauer spectra in the (a) prepared Fe_3_C sample with a large intensity sextet (purple) identified as θ-Fe_3_C and (b) synthesized Fe_5_C_2_ powder with three intense sextets of χ-Fe_5_C_2_ (I maroon, II red, III salmon).

The fitted Mössbauer spectrum of the Fe_3_C sample (Figure 2a) shows a sextuplet with an isomer shift (IS) of 0.19 mm s^–1^, and a hyperfine field of 20.8 T. θ-Fe_3_C is identified as the major spectral contributor (67%).^51^ Additionally, a metallic Fe sextuplet (IS = −0.004 mm s^–1^, 33 T) was clearly observed and is in agreement with the sharp peak at 53.3° in the Fe_3_C diffractogram. A doublet indicates a quadrupole peak splitting, which means the absence of magnetic field spin coupling. This indicates the presence of (super)paramagnetic Fe^3+^ nanostructures. It is difficult to allocate the specific Fe^3+^ phase, as multiple subdoublets can be superimposed in one doublet.^52^ However, the low intensity XRD peaks of Fe_2_O_3_ suggests that the doublet contains mostly nanostructured Fe_3_O_4_. The presumably low quantities of Fe_2_O_3_ are covered by a sextet (IS = 0.31 mm s^–1^, 49.3 T); therefore, it is unlikely that Fe_2_O_3_ has a spectral contribution in the doublet. Three sextuplets (IS = 0.27, 0.21, and 0.16 mm s^–1^ with B_hyp_ = 21.7, 18.1, and 10.3 T) covered 78% of the spectral area in Figure 2b, which were attributed to the three iron lattice sites in the Fe_5_C_2_ crystal structure.^53,54^ Fe_3_O_4_ has a small spectral contribution located in the outer spectrum with an octahedral (IS = 0.28 mm s^–1^, 49.1 T) and a tetrahedral site (IS = 0.71 mm s^–1^, 46 T).^55^ Fe_2_O_3_ was not identified in the Fe_5_C_2_ diffractrogram, which again suggests that the doublet is nanostructured Fe_3_O_4_. In conclusion, the Mössbauer data confirms the synthesis of the intended Fe_3_C and Fe_5_C_2_ compounds with limited amounts of iron and iron oxide species. The remainder of the Mössbauer data is summarized in Table S2.

**Figure 3:**
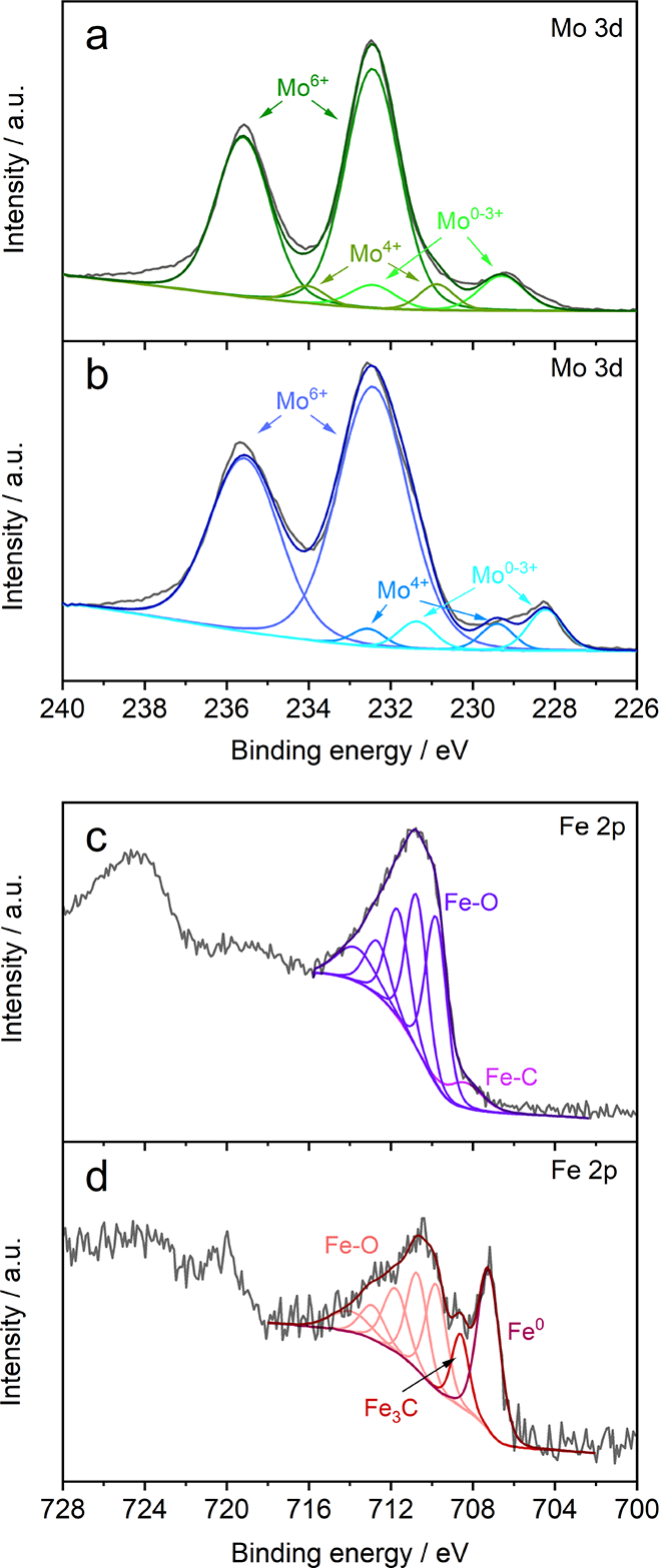
X-ray photoelectron spectroscopy Mo 3d and Fe 2p spectra with deconvoluted peaks in (a) Mo_2_C NS (green) and (b) Mo_2_C NP (blue), (c) orthorhombic Fe_3_C (purple), and (d) Fe_5_C_2_ (red).

Mössbauer spectroscopy and XRD give information about the bulk phase of the material. XPS is surface sensitive and gives information regarding the surface phase and composition. The Mo_2_C XPS survey scans (Figure S6a,b) contain peaks of Mo 3d, Mo 3p, C 1s and O 1s, from which the high-resolution scans of the Mo 3d photoelectrons (Figure 3a,b) were deconvoluted to identify different Mo oxidation states. The Mo 3d orbital has a spin–orbit Mo 3d_5/2_-Mo 3d_3/2_ doublet with a 3/2 peak intensity ratio that is separated by a binding energy of 3.15 eV. The full width at half-maximum (FWHM) was kept constant for each doublet during the deconvolution process. It is often ambiguous to assign an oxidation state to Mo_2_C; therefore, it is often denoted in an aggregated term as Mo^0–3+^.^56^ The presence of Mo_2_C surface bonded species was confirmed by the small Mo 3d_5/2_ peaks at 229.3 and 228.2 eV for both Mo_2_C NS and Mo_2_C NP, respectively.^34,57^ Other peaks at binding energies 232.5 and 235.5 eV for both Mo-carbide materials are identified as MoO_3_ and must be solely present in the thin surface layers as MoO_3_ was not identified in the diffractograms. These spontaneously formed metal oxide surface layers are inevitable due to exposure to ambient air. Post-mortem XPS analyses confirmed that the majority of the surface layer was Mo_2_C.^23,58^ This suggests that the Mo-oxide species are reduced during electrochemical reduction. Moreover, it is expected that the trans-passive Mo-oxide layers are not stable in alkaline conditions and form soluble MoO_4_^2–^ even at moderate reduction potentials.^59,60^

The Fe 2p^3/2^ peak was used to identify different Fe oxidation states. The broad peak between 714 and 709 eV contains a complex convolution of multiple subpentuplets of Fe_2_O_3_, Fe_3_O_4_, and FeOOH species, which all overlap in this region. We fitted one pentuplet as a general Fe-oxide term as indicated in Figure 3c,d by taking XPS reference data such as FWHM, relative peak area, and binding energies from Biesinger et al.^61^ For iron carbide, the majority of the surface is covered with a thin Fe-oxide layer. According to the Pourbaix diagram for Fe, this oxide-layer is reduced by applying mild reduction potentials.^62^ The presence of a single peak at 708.4 eV for Fe_3_C and 708.6 eV for Fe_5_C_2_ is identified as the Fe carbide phase. Only Fe_5_C_2_ has an additional sharp metallic Fe peak at 707.2 eV. The low signal-to-noise ratio for the Fe_5_C_2_ Fe 2p spectra indicates a low Fe quantity (<1 at%), which is also reflected in a low intensity Fe oxide peak in the O 1s spectra (Figure S7e). From a depth profiling test (Figure S8), it becomes clear that the Fe 2p signal increases with a longer etching time, while the intensities of the O 1s and N 1s spectra decreases. This indicates that the top surface layer is covered with adventitious species due to atmospheric exposure.

The elemental Mo and Fe content in all metal carbides were analyzed by ICP-OES and are summarized in Table S3. The ICP-OES results revealed that the bulk concentrations of Mo and Fe are significantly higher with respect to the surface concentrations estimated by XPS. This suggests that the surface adsorption of advantageous species by air exposure is not only observed for Fe_5_C_2_ but also for the other metal carbides.

**Figure 4:**
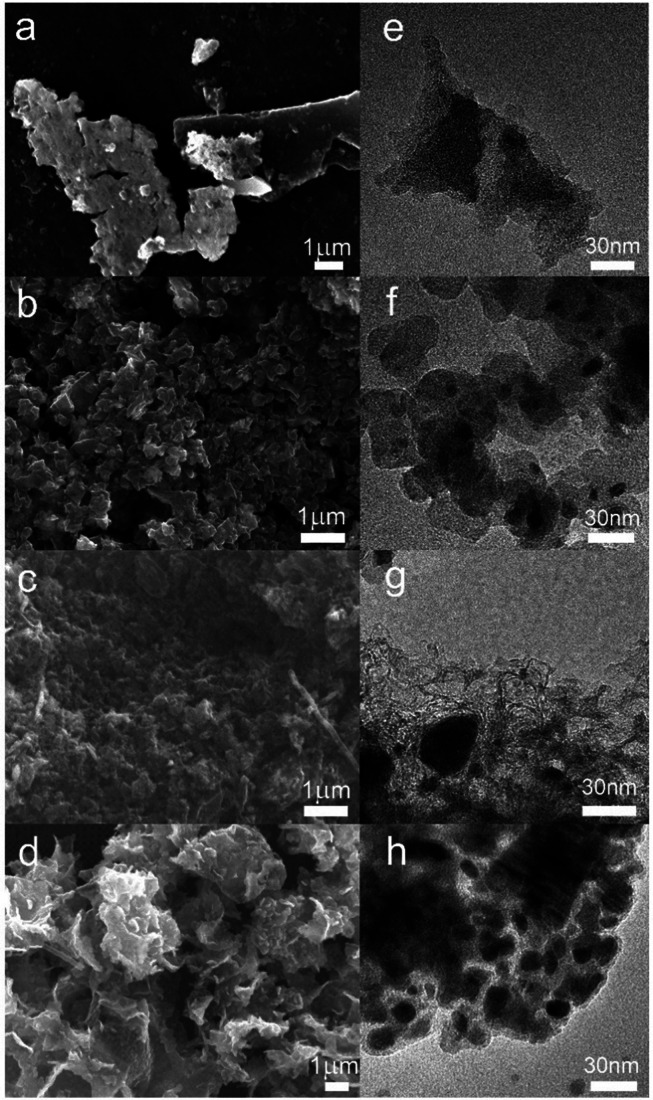
Scanning and transmission electron micrographs of (a, e) Mo_2_C NS, (b, f) Mo_2_C NP on a carbon support, (c, g) Fe_3_C, and (d, h) Fe_5_C_2_.

The Mo_2_C NS are clearly visible in Figure 4a,e and confirm a successful synthesis. TEM imaging (Figure S9a) reveals that a relatively large proportion of the sample consists of undecorated carbon nanosheets. This explains why the majority of the surface composition, analyzed by XPS, is predominantly carbon (Table S4). The existence of Mo_2_C nanodots (<20 nm), as proposed by Wang and coworkers, was not observed in our TEM analysis.^23^ Despite the magnification limitations of the low-resolution TEM, distinguished nanoparticles up to 5–10 nm were detectable in other metal carbide samples, indicating that Mo_2_C nanodots of the order 10–20 nm should be visible. In Figure 4e and Figure S9b, regions with a higher contrast indicate a layer of aggregated Mo_2_C, with an average crystallite size (*D*_XRD_) of 35 nm. The SEM–EDX results (Figures S10 and S11) support this observation and show that the carbon sheet is indeed covered with a nanocrystalline layer of Mo_2_C.

The successful synthesis of Mo_2_C NP on a carbon support was confirmed by TEM (Figure 4f and Figure S12). The particle size, *D*_TEM_, was distributed between 10 and 50 nm, which is in agreement with the average crystallite size (*D*_XRD_ = 21 nm). The SEM images in Figure 4b and Figure S13 show a mesoporous morphology with a large surface area. The overall Mo_2_C surface distribution is homogeneous as was confirmed by the SEM–EDX mapping (Figure S14). The carbon precursor in combination with the inert SiO_2_ nanoparticles stimulates the spherical growth of nanosized Fe_3_C particles and prevents it from forming larger aggregates. Most Fe_3_C particles were between 40 and 60 nm. The Fe_3_C sample contained nanosized hollow features as visible in Figure 4c,g and confirmed the successful removal of SiO_2_ during the 1 M KOH treatment. The absence of the SiO_2_ nanoparticles after the treatment was further supported by Fourier transform infrared spectroscopy (Figure S15).

Small and isolated Fe_5_C_2_ spherical nanoparticles with a narrow size distribution are observed in Figure 4d and Figure S16 (*D*_TEM_ is 5–35 nm and *D*_XRD_ is 11 nm). This highlights that PVP successfully stabilizes the nanoparticles from agglomeration during the carbothermal reduction of Fe(CO)_5_. The material has a microporous structure (Figure S17b) with a high surface area because of the polymeric nature of the support. In contrast to the low Fe content measured in the first ∼10 nm-thick surface layer, well distributed and significant Fe concentrations were detected in the bulk surface layers (∼1 μm) by SEM–EDX mapping (Figure S18), which supports the XPS depth profiling and ICP-OES results (Figure S8).

### 3.2 Electrochemical Characterization

The current-potential (*I*–*V*) relationship of each material was investigated by executing multiple CV cycles at a scan rate of 20 mV·s^–1^ in a N_2_ presaturated electrolyte to estimate the onset potential and an expected potential window for the NRR. A possible pH dependency on the Mo_2_C activity of the NRR was investigated by executing CV in 0.05 M H_2_SO_4_ (pH = 1), 0.5 M Li_2_SO_4_ (pH = 8.3), 0.1 M KOH (pH 13), and 1 M KOH (pH 14). The stability of Fe-carbides in acidic-to-neutral conditions is low as the material tends to dissolve.^63^ Therefore, we decided to only use 0.1 M and 1 M KOH for the evaluation of the Fe-carbides. Mo_2_C is generally stable in both acidic and alkaline environments, allowing CV measurements in all electrolytes.^58,64^ The uncompensated resistance (*R*_u_), measured with open-circuit electrochemical impedance spectroscopy was consistent for each material tested and ranged between 25 and 30 Ω for 0.1 M KOH, 3 and 4 Ω for 1 M KOH, 12 and 13 Ω for 0.5 M Li_2_SO_4_, and 24 Ω for 0.05 M H_2_SO_4_ (Figure S19). These quantities for *R*_u_ are below the acceptable range of the *R*_u_ compensator used for all electrochemical measurements.

**Figure 5:**
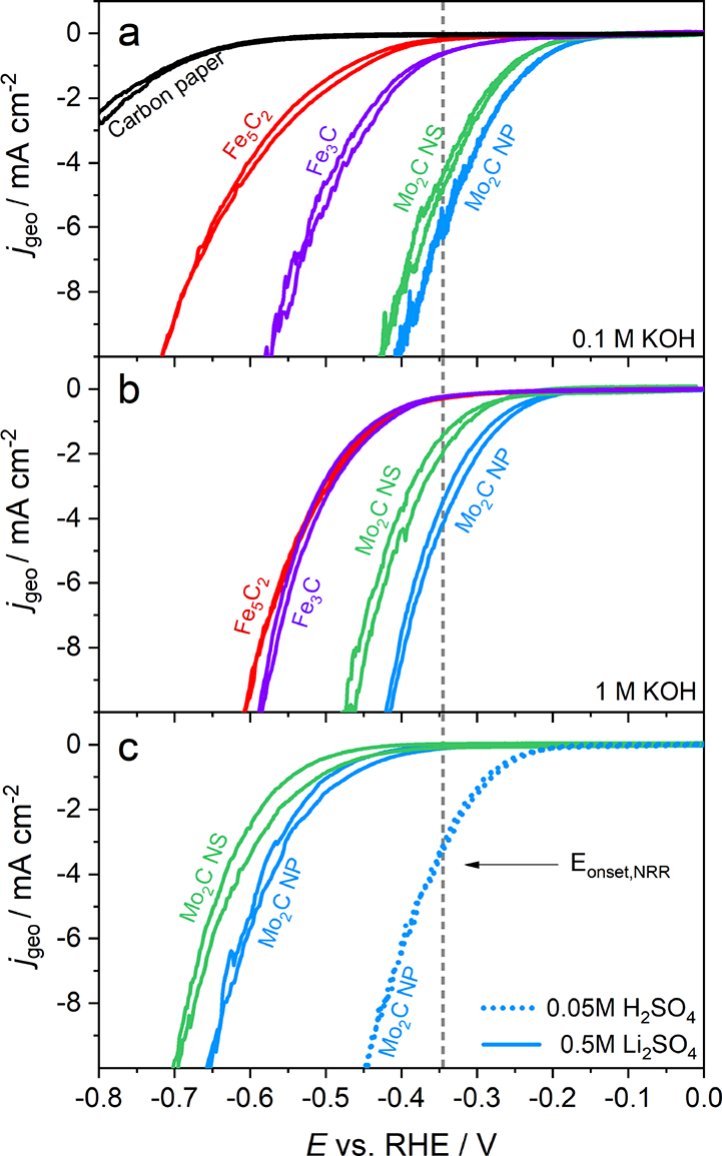
Cyclic voltammograms (6th cycle) at 20 mV·s^–1^ for Mo_2_C NS (green), Mo_2_C NP (blue), Fe_3_C (purple), Fe_5_C_2_ (red), and carbon paper (black) in (a) 0.1 M KOH, (b) 1 M KOH, (c) 0.5 M Li_2_SO_4_, and 0.05 M H_2_SO_4_. The gray dotted line at −0.345 V vs RHE represents the theoretically estimated onset potential for the NRR.

Figure 5 shows that all metal carbide *I*–*V* relationships in acidic, neutral, and alkaline conditions display an increase in current density at increasingly more negative potentials, characteristic for an HER *I*–*V* profile. Other distinctive reduction peaks that might be identified as the NRR were not observed in the voltammetry measurements. In addition to this, there was no indication of a metal oxide reduction peak within the examined potential window, which suggests that the metal oxide surface layer is removed immediately. Mo_2_C NP reaches higher current densities compared to Mo_2_C NS at all pH values, which could be explained by a larger electrochemical surface area due to the mesoporous structure of Mo_2_C NP. Another explanation might be the higher Mo_2_C loading content in Mo_2_C NP, since the ICP-OES analysis resulted in a higher concentration of elemental Mo in Mo_2_C NP.

The onset potential is used to indicate the minimum activation potential of a redox reaction in cyclic voltammograms.^65^ A theoretical approximation of the NRR onset potential in alkaline media can be calculated using the equilibrium potential and the activation overpotential. First-principles density functional theory calculations suggest that the minimum overpotential for the NRR is approximately −0.4 V, due to scaling relationships between intermediates.^2,66^ The NRR equilibrium potential was calculated by equilibrium thermodynamics and is 0.054 V vs RHE, which results in a minimum required onset potential (*E*_*o*nset, NRR_) of −0.35 V vs RHE. Here, we estimated the experimental *E*_onset_ for different pH values by plotting the first derivative of the voltammogram (d*j*/d*E*) versus the applied potential (Figure S20). The lift-off point where the slope of the d*j*/d*E* curve starts to increase is set as the *E*_onset_.

For 0.1 M and 1 M KOH, we do not see clear evidence for an alkaline pH effect for Mo_2_C materials as both Mo_2_C NP and NS have a similar *I*–*V* curve at both KOH concentrations. The onset potential for Mo_2_C NP of −0.11 V vs RHE is similar for both electrolytes and is in agreement with earlier observations.^64^ Moreover, the onset potentials for Fe_3_C and Fe_5_C_2_ are also similar and varied between −0.22 and −0.23 V vs RHE for both 0.1 M and 1 M KOH. The *I*–*V* relationship in Figure 5c for Mo_2_C in 0.5 M Li_2_SO_4_ is remarkably different showing a more negative onset potential of −0.28 V vs RHE and −0.32 V vs RHE for Mo_2_C NP and NS, respectively. As a consequence, the activation overpotential at −10 mA·cm^–2^ for Mo_2_C NP is −0.25 V lower than in alkaline conditions, which can be related to the low availability of either protons or hydroxide ions. At acidic conditions (pH = 1), Mo_2_C NP displays a similar *I*–*V* relationship with respect to alkaline conditions, which highlights the unique properties of Mo_2_C showing similar catalytic activity in both acidic and alkaline conditions.^64^ However, the onset potential is slightly more negative (−0.17 V vs RHE), indicating that the catalyst is more active in alkaline conditions.

*E*_onset_ for Mo_2_C is above the theoretically estimated threshold in both acidic and alkaline pH, where the current density *E*_onset,NRR_ is roughly −4 mA·cm^–2^ for Mo_2_C NP. From this analysis, it is unlikely that the NRR is a dominant contributor to the *I*–*V* profile of Mo_2_C because the HER kinetics are more facile in these conditions. Interestingly, *E*_onset_ for Mo_2_C in 0.5 M Li_2_SO_4_ is below *E*_onset,NRR_ and suggests that operating at near-neutral conditions might be ideal for the NRR. It is important to note that Cheng and coworkers reported high NH_3_ yields with Mo_2_C NS using the same electrolyte.^23^ For Fe_3_C and Fe_5_C_2_, the majority of the *I*–*V* profile exceeds *E*_onset,NRR_, suggesting that both iron carbides might be promising catalysts for the NRR.

The NRR activity of the metal carbides was qualitatively screened by measuring the ammonia concentration after executing 40 scans of cyclic voltammetry with a scan rate of 20 mV s^–1^. The results are summarized in Figure S21 and show that NH_3_ concentrations for Mo_2_C in acidic-neutral pH is close to the detection limit <30 ppb, while levels up to 100 ppb were observed in alkaline conditions. Operating at alkaline conditions is therefore more beneficial for studying the NRR, and subsequently, the main electrochemical experiments herein were performed in alkaline conditions.

To ultimately verify Mo- and Fe-carbides as conceivable NRR catalysts, a series of 2 h chronoamperometry (CA) measurements were performed at five different potentials in a N_2_-saturated 0.1 M KOH electrolyte. The current densities for the metal carbides are stable in alkaline conditions as illustrated in Figure S22. As a comparison, two additional CA measurements in Ar-saturated electrolytes were performed with Mo_2_C, which gave slightly higher current densities for two potentials. The difference in current density is however rather small and can be caused by slight variations in the experiments. This observation was also made elsewhere,^28^ questioning the reliability of N_2_ vs Ar voltammetry and CA experiments as an initial indicator for successful dinitrogen reduction.

### 3.3 NRR Measurements

CA measurements were used to further assess the activity toward the NRR. After each CA, aliquots of both catholyte and anolyte were taken from the cell for further quantification of NH_3_ and NO_2_^–^. The amount of quantified NH_3_ after each experiment varied between 37 and 123 ppb with no particular trend linking ammonia concentration and applied potential over time. A 2 h open circuit potential (OCV) test with a N_2_-saturated electrolyte was used to obtain insights on the amount of impurities coming from either the feed gas stream or surface adsorbed species inside the cell. The OCV results for Mo_2_C reveal a similar NH_3_ concentration as obtained with the chronoamperometry experiments. The impact of feed gas impurities can be excluded due to the installed certified gas filter (<100 ppt) in front of the cell. It is more likely that adsorbed NH_3_ in the cell components is released during the OCV experiments and inevitably during the NRR measurements. Long term CA experiments with an Ar-saturated electrolyte are useful to study the possible release of N-impurities from the catalyst and other cell components exposed to the electrolyte under electrochemical conditions. For Mo_2_C, three CA experiments with Ar-saturated electrolyte at −0.20, −0.31, and −0.44 V vs RHE resulted in a somewhat lower NH_3_ content (80, 53, and 60 ppb) compared to experiments with N_2_-saturated electrolytes. Again, it is deemed unlikely that purified Ar (and N_2_) introduces feed gas impurities. Therefore, this observation suggests that the Ar gas flowing through the electrolyte stripped a small part of the dissolved NH_3_ from the electrolyte. Nevertheless, both the N_2_ OCV and Ar CA experiments indicate that the majority of the quantified NH_3_ is not from the NRR but originates from contaminations. Additional control experiments with ^15^N_2_-labeled gas were not performed since the observed NH_3_ concentrations were below or approximating the background level.

Small quantities of NO_2_^–^ were detected after all CA experiments, suggesting that a part of the quantified NH_3_ potentially stems from NO_*x*_ reduction. Jiao and coworkers observed that the electrochemical reduction of NO_*x*_ forms multiple N-products, such as ammonia, hydroxylamine, N_2_, and N_2_O depending on the transition metal.^67^ Pt is more selective toward NH_3_, which was also supported by Koper and coworkers who made a similar observation for NO_3_^–^ electroreduction on Pt.^68^ Mo_2_C has similar noble metal-like properties as platinum;^69^ therefore, it is reasonable to assume that NO_*x*_ species are reduced to ammonia at the investigated potentials. Fe_3_C is also an efficient nitrate reduction electrocatalyst, where a previous study reported faradaic efficiencies (FE) higher than 90% to NH_3_ at moderate reduction potentials.^70^

Despite the thorough cleaning efforts for every part of the cell, a well-established background level of both NH_3_ and NO_2_^–^ was always observed after each experiment. We decided to analyze the removal efficiencies of our cleaning methods (elaborated in the caption of Figure S23) and found that NH_3_ was sufficiently removed by simply rinsing with water. Surprisingly, significant quantities of released NO_2_^–^ were detected that originated from the Celgard 3401 membrane, carbon paper, and Pt foil. This is a valuable observation, as two previous studies advised substituting the Nafion membrane with a microporous Celgard membrane to reduce NH_3_ contaminations.^15,45^ Our results indicate that NO_2_^–^ is not only a surface adsorbed species but is also present in the inner membrane and carbon paper structure and is problematic to remove. Generally, the amount of released NO_2_^–^ depends mainly on the exposed surface area, meaning that it can be lowered significantly by optimizing the cell design. Investigating the origin of the observed NO_*x*_ impurities is out of the scope of the present work and will be addressed in an upcoming study.^46^

### 3.4 Literature Comparison

**Figure 6:**
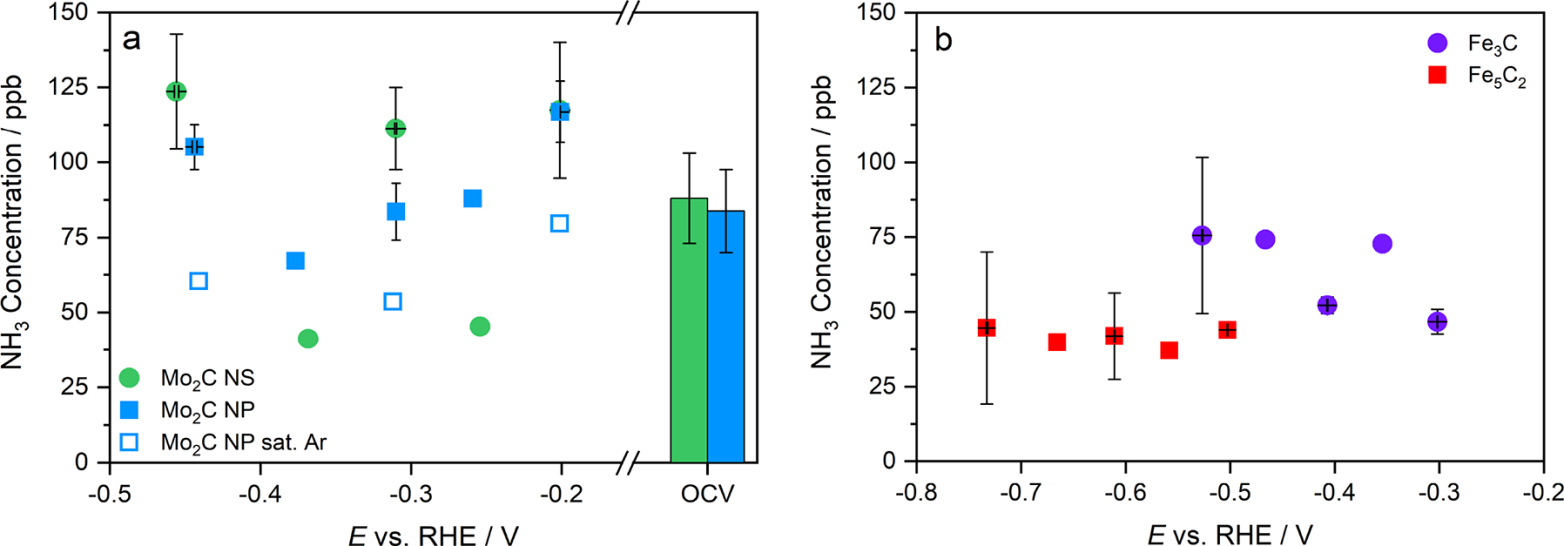
NH_3_ concentration measured from the electrolyte after two hour CA experiments in 0.1 M KOH. (a) Mo_2_C NS (green, spherical) and Mo_2_C NP in N_2_ (blue, rectangular) and Ar (open rectangular). (b) Fe_3_C (purple, spherical) and Fe_5_C_2_ (red, rectangular). The data points with the error bars were done in duplicates.

**Figure 7:**
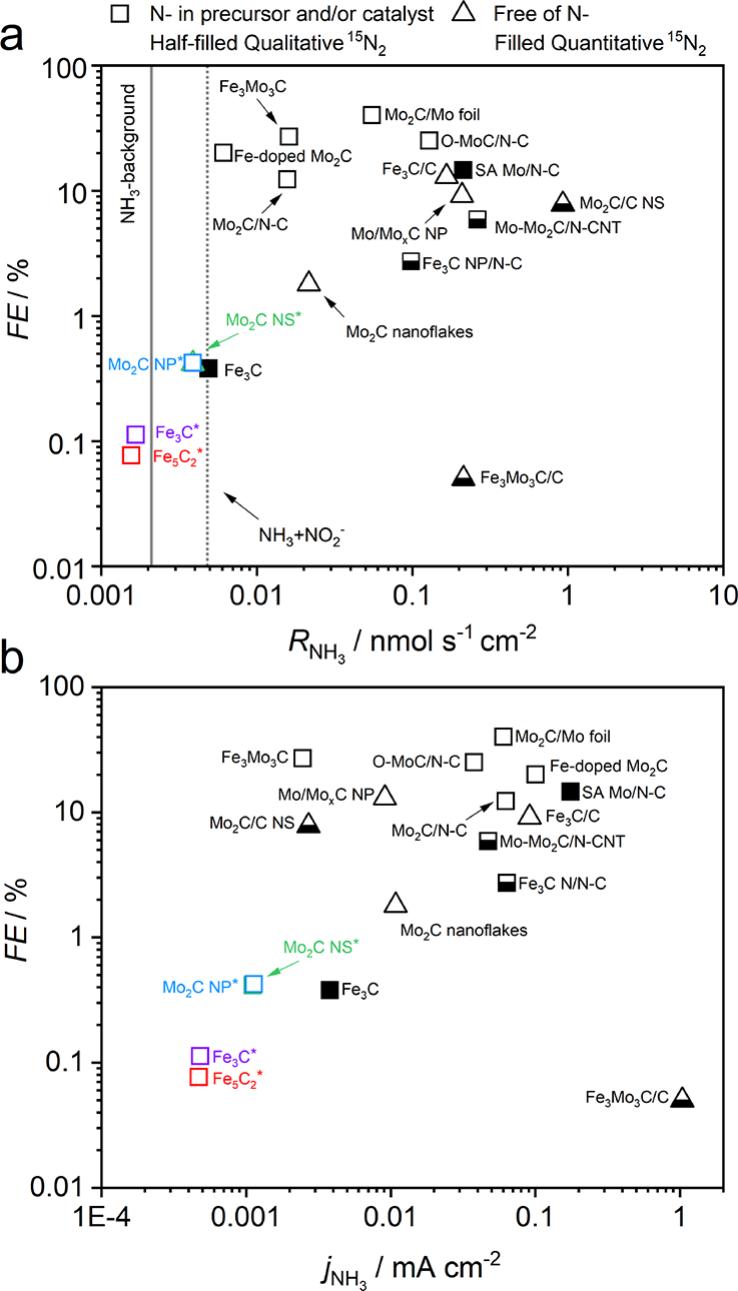
Literature overview of recently published NRR studies using Mo- and Fe-carbides as electrocatalysts differentiated by either high N-source in the support or used during synthesis (square) and free of N-source (triangular). The symbols are filled in case of quantitative ^15^N_2_-labeled experiments and half-filled if analyzed qualitatively. Our own results are included as Mo_2_C NS* (green), Mo_2_C NP* (blue), Fe_3_C* (purple), and Fe_5_C_2_* (red). (a) Faradaic efficiency vs NH_3_ yield with thick line (gray) indicates the estimated NH_3_ background level and the dotted line presents a hypothetical background level including the measured NO_2_^–^ from Figure S24. (b) Faradaic efficiency vs *j*_NH_3__. More details regarding the literature studies included in the figure can be found in Table S4.

Previous studies using Mo- and Fe-carbides as NRR electrocatalysts are shown in a comparative overview (Figure 7a), including our own observations. It becomes clear that both our Fe_3_C and Fe_5_C_2_ quantified NH_3_ yields are within the NH_3_ background level. The Mo_2_C catalysts exceed this threshold slightly, but with a significant NO_*x*_ background, it becomes unlikely that any nitrogen reduction to NH_3_ occurred. The study of Cheng and coworkers outperformed our Mo_2_C NS, observing a 240 times higher NH_3_ yield.^23^ This motivated us to execute a direct comparison by increasing the catalyst loading to 3 mg·cm^–2^ and using 0.5 M Li_2_SO_4_. The chronoamperometry measurements were comparable, but our NH_3_ concentrations were below 100 ppb and close to the earlier defined background level as displayed in Figure 6. This is additional proof that Mo_2_C cannot be perceived as a promising NRR catalyst.

The majority of the earlier published literature observed orders of magnitude higher yields and FEs compared to this work. Firstly, all the literature studies shown in the overview did not quantify or consider NO_*x*_ as an influential factor on their measured NH_3_ content. Secondly, the impurities in the feed gas stream were not removed by the installment of a certified gas filter. This is especially important when performing ^15^N_2_-isotope labeled experiments as traces of ^15^N-labeled impurities (^15^NH_3_ and ^15^NO_*x*_) have been identified in several ^15^N_2_-gas bottles.^15,25,71,72^ Before using Li-based electrolytes for NRR experiments, Li-salts must be thermally annealed at 800 °C under inert conditions to remove trace levels (>1 ppm) of NO_*x*_^–^ impurities.^47^ We followed this procedure, while others, including Cheng et al., did not consider this extraneous source of impurities, and this might be one of the main factors contributing to their high NH_3_ yields.^23,31,36^ As final point, the Nafion membrane commonly applied in these studies is known for the uptake and release of NH_3_ during electrochemical experiments.^15^ Substituting the Nafion membrane with another membrane is not straightforward as we detected a significant amount of NO_2_^–^ in the microporous membranes (Celgard 3401), but selecting a suitable treatment method is advised.^73^

Control experiments become even more essential when catalysts have a high N-content, such as metal nitrides, N-doped supports, or leftover NO_*x*_/NH_3_ traces from the catalyst synthesis. Evidence was found that for catalysts with a high N content, such as VN and Nb_4_N_5_, the decomposition of the N-atomic lattice in acidic media released significant amounts of NH_4_^+^ during the initial stages of the electrochemical experiment.^28^ Similar observations were also reported for Mo_2_N.^30^ Additionally, several commercially available metal oxide powders, such as Fe_2_O_3_ and Bi_2_O_3_, released a large amount of NO_*x*_ impurities. This eventually led to the retraction of a study, as it was proven that the origin of observed NH_3_ was from NO_*x*_ reduction and not the NRR.^26,74^ Therefore, we decided to analyze the N-content of all four materials by XPS and UV–Vis spectroscopy (method described in the SI). The N 1s spectra of Mo_2_C could not be identified because of overlapping peak features with the Mo 3p orbital. Nevertheless, the absence of N KLL Auger peaks in both Mo_2_C NS and NP XPS surveys (Figure S6a,b) indicate that the N-content might be negligible. Figure S7c,f shows two distinct N 1s peaks for Fe_3_C (398.3 and 399.9 eV) and Fe_5_C_2_ (398.9 and 400.1 eV), suggesting pyridinic N–C and pyrrolic N–C bonds from the precursor (4.5-dicyanoimidazole and PVP).^75^ Nevertheless, the samples were exposed to air before XPS analysis, indicating that the peaks could be also from adventitious N species, such as −NH_2_, which have similar binding energies.^75−77^ It is therefore challenging to assign these peaks to a specific N-functional group. From the spectrophotometric analysis (Figure S25), directly performed after the material synthesis, it becomes clear that NH_3_ impurities from an unidentifiable source were present in all catalysts (Mo_2_C NS = 8.9 μmol_NH3_ g_cat_^–1^ , Mo_2_C NP = 16.5 μmol_NH3_ g_cat_^–1^ , Fe_3_C = 21.9 μmol_NH3_ g_cat_^–1^, Fe_5_C_2_ = 4.5 μmol_NH3_ g_cat_^–1^). This effect was suppressed by using a low catalyst loading (0.06 mg) for each experiment. In the case of the most contaminated sample, the expected release of NH_3_ from 0.06 mg Fe_3_C is limited to a negligible 1.3 nmol. Nevertheless, the NRR measurements performed with 3 mg Mo_2_C NS did not result in an increase in the NH_3_ concentration. It remains unlikely that impurities in the catalyst resulted in exceptionally high NH_3_ yield reported by Cheng et al.^23^ This suggests that other factors lead to their positive result.

The NH_3_ partial current density, *j*_NH_3__, is a useful performance indicator, wherein cases with *j*_NH_3__ smaller than 100 μA cm^–1^ are too low to be promising. From a back-of-the-envelope calculation, we estimated that the NH_3_ concentration at *j*_NH_3__=100 μA cm^–1^ is in the 1 ppm order of magnitude range assuming typical parameters, such as *A*_WE_ = 1 cm^2^, *V*_catholyte_ = 20 mL, and *t*_CP_ = 1 h. These levels of NH_3_ can easily be reached when the earlier mentioned sources of contamination are not identified or even considered. This has implications on the reliability and usefulness of reporting the FE, wherein the focus should be initially on *j*_NH_3__ or the NH_3_ yield rate. From Figure 7b, it becomes clear that most literature studies did not exceed 100 μA cm^–1^, while a FE > 20% was reported (see Table S4). Therefore, we suggest that future publications explicitly report the NH_3_ partial current density as the main catalyst performance indicator.

Interestingly, the role of metal carbides is also under debate for hydrazine oxidation.^78^ Fe–N–C catalysts are common used catalysts for this reaction and contain iron carbides because of the high temperature pyrolysis required for the synthesis. Early studies claimed that Fe_3_C plays an active role in the reaction,^79,80^ while a recent study revealed the true role of Fe_3_C by executing a rigorous comparison study between Fe–N–C materials with different amounts of Fe_3_C.^78^ This approach led to the conclusion that Fe_3_C is mostly inactive for hydrazine oxidation, and should be removed by nonoxidizing acid solutions. This is yet another example of how a rigorous and well-designed experimental procedure can aid in clarifying the activity of electrocatalysts for reactions in the nitrogen cycle.

## 4 Conclusions

Nanostructured molybdenum carbide and iron carbide were reported earlier as promising electrochemical nitrogen reduction catalysts. In this study, the NRR activity of both molybdenum and iron carbide materials were reassessed with the implementation of a strict experimental protocol that allowed us to reduce the effects of extraneous impurities to a bare minimum and identify false positives. The successful synthesis of nanostructured Mo_2_C, Fe_3_C, and Fe_5_C_2_ was confirmed by X-ray diffraction, scanning and transmission electron microscopy, and X-ray photoelectron and Mössbauer spectroscopy. The current–potential relationship of the metal carbides is characteristic for the HER, where the current increases with increasing negative overpotential. Moreover, specific reduction peaks that could be related to the NRR were not identified. NH_3_ quantification was done after 40 scans of cyclic voltammetry, where we indeed measured NH_3_ (50–100 ppb) for both Mo_2_C and Fe_5_C_2_ in alkaline conditions. To further assess the NRR catalytic activity of molybdenum and iron carbides, we performed a series of 2 h chronoamperometry measurements at different potentials in N_2_-saturated 0.1 M KOH. For Mo_2_C NP and NS, the NH_3_ concentration was between 41 and 124 ppb, exceeding the NH_3_ background level (84–88 ppb) for potentials at −0.2, −0.31, and −0.46 V vs RHE. We noticed that the yield earlier reported by Cheng et al. was considerable higher than measured with our Mo_2_C NS.^23^ A direct comparison by performing chronoamperometry experiments with an increased loading (3 mg·cm^–2^) and 0.5 Li_2_SO_4_ did not result in elevated NH_3_ concentrations. This is additional proof that Mo_2_C cannot be conceived as a promising NRR catalyst. The NO_*x*_ content after the NRR, Ar, and OCV blank tests revealed NO_2_^–^ concentrations in the same order of magnitude (55–122 ppb). This implies that NH_3_ arises from NO_2_^–^ reduction and not from the NRR. These NO_2_^–^ impurities originated from the Celgard membrane, since we found that the membrane, even after rinsing excessively with water, released a considerable amount of NO_2_^–^ impurities (109 ± 31 ppb). This emphasizes the importance of NO_*x*_ monitoring, which is often overlooked in the literature and might result in a false positive. The quantified NH_3_ from the iron carbide catalysts did not exceed the NH_3_ background level, indicating that these materials are not active for the NRR. With our experimental approach, we succeeded in establishing a minimized and reproducible background level that allowed us to critically assess promising NRR catalysts. We believe that our methods and detailed analysis will equip researchers entering the field with clear guidelines to perform NRR experiments in a more reliable manner.

## Supporting Information Available

Additional information on the experimental setup, UV–vis calibration curves, XRD analysis, Mössbauer data, XPS, SEM–EDX, TEM, ICP-OES, FT-IR, electrochemical data, impurity assessment, and a literature summary (PDF)
