# Supplementary material for: Revisiting the Electrochemical Nitrogen Reduction on Molybdenum and Iron Carbides: Promising Catalysts or False Positives?
Source: ACS Catal. 2023 Jan 12;13(3):1649–61. doi: 10.1021/acscatal.2c04491 (PMC9903294; doi:10.1021/acscatal.2c04491)
Supplement: Supplementary file 1 — cs2c04491_si_001.pdf [file cs2c04491_si_001.pdf]

Supporting Information

Revisiting the Electrochemical Nitrogen Reduction on Molybdenum and Iron Carbides: Promising Catalysts or False Positives?

Boaz Izelaar, Davide Ripepi[†], Simone Asperti, Iulian A. Dugulan[‡], Ruud W.A. Hendrikx[⊥], Amarante J. Böttger[⊥], Fokko M. Mulder[†], Ruud Kortlever[*]

Large Scale Energy Storage, Process and Energy Department, Faculty of Mechanical, Maritime and Materials Engineering, Delft University of Technology, 2628 CB Delft, The Netherlands

[†]Materials for Energy Conversion and Storage, Chemical Engineering Department, Faculty of Applied Sciences, Delft University of Technology, 2629 HZ Delft, The Netherlands

[‡]Radiation Science and Technology Department, Faculty of Applied Sciences, Delft University of Technology, 2629 HZ Delft, The Netherlands

[⊥]Surface and Interface Engineering, Materials Science and Engineering Department, Faculty of Mechanical, Maritime and Materials Engineering, Delft University of Technology, 2628 CB Delft, The Netherlands

Corresponding Author; E-mail: R.Kortlever@tudelft.nl

Supporting Figures

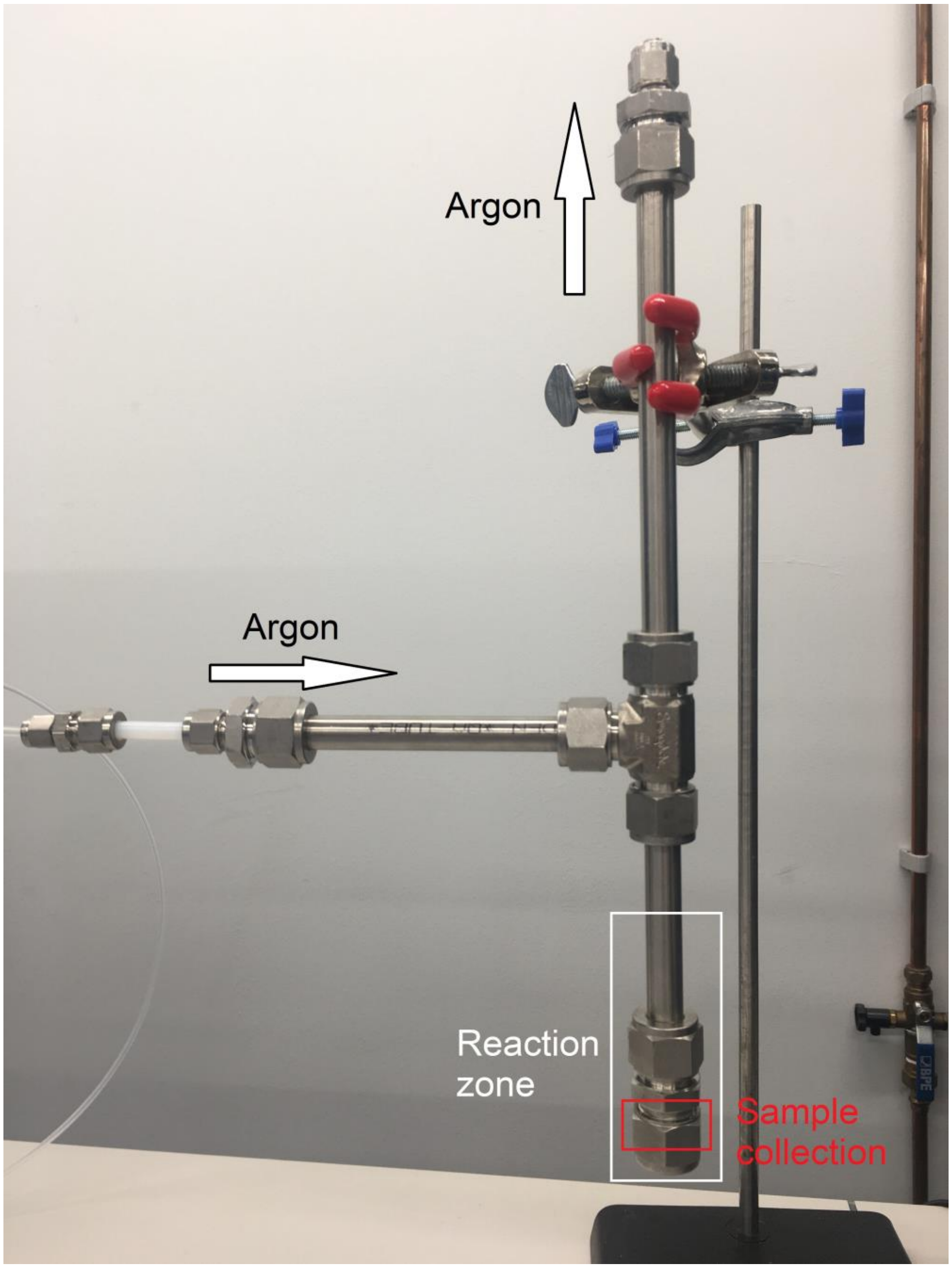

Figure S1. Homemade gas-tight reaction chamber from Swagelock stainless steel tubes and adapters for the synthesis of χ-Fe_5C_2. Polyvinylpyrrolidone was mixed with iron(0) pentacarbonyl in the reaction zone as indicated. After the thermal-decomposition process, the reactor was depressurized and the sample was collected from the bottom part of the reactor.

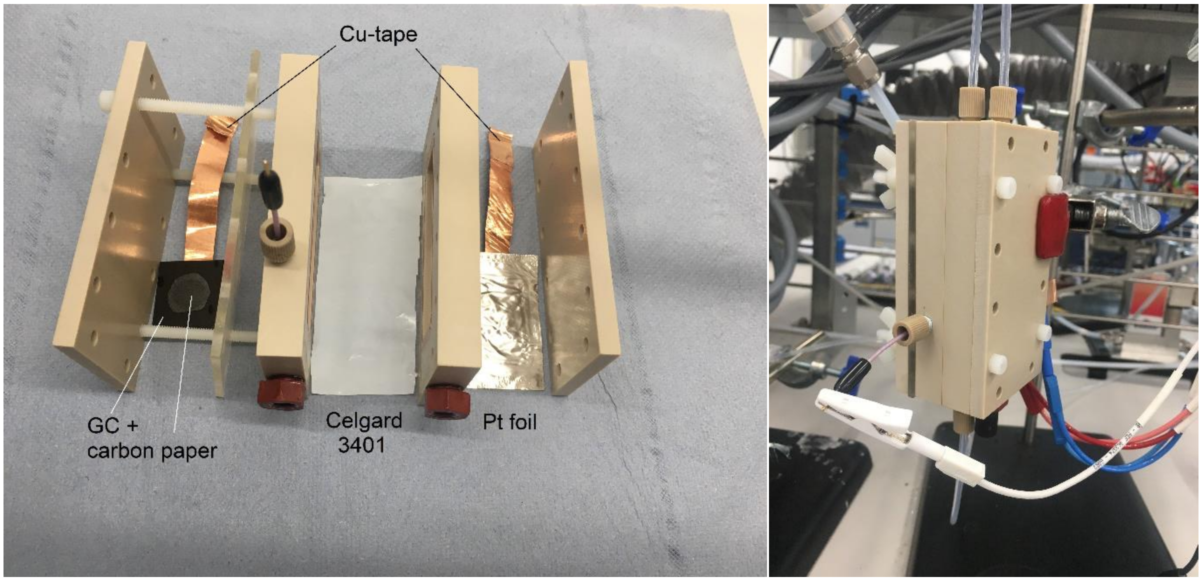

Figure S2. (left) Photograph of the PEEK cell body adapted from the Jaramillo group and its components.[1] (right) The cell assembled and connected to the potentiostat.

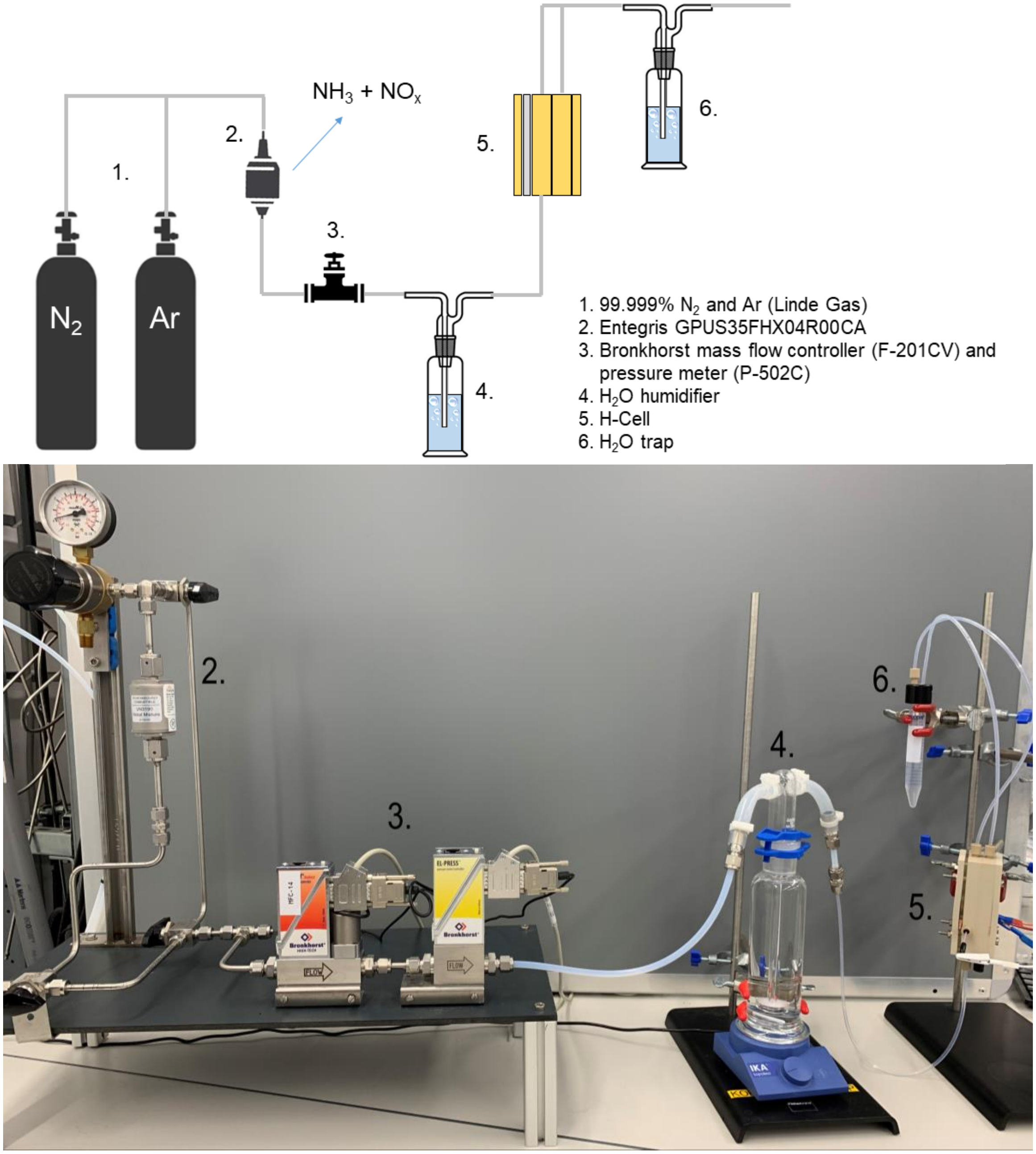

Figure S3. Photograph of the experimental setup including enumerated labels (bottom) and an explanatory schematic (top).

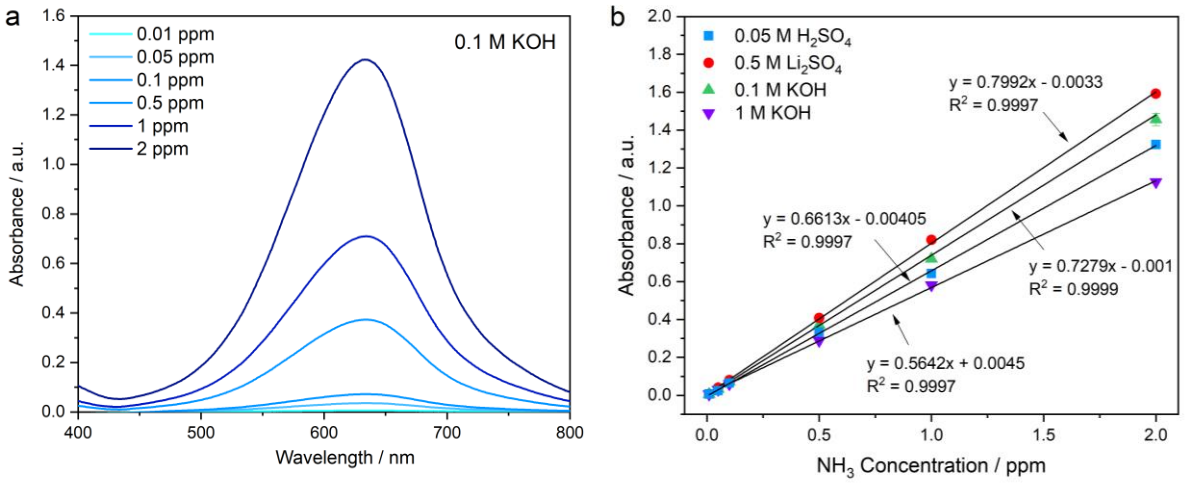

Figure S4. (a) UV-Vis spectra of 0 – 2 ppm NH_3 concentrations in 0.1 M KOH with a maximum absorbance at 633 nm, and is also representative for other pH. (b) NH_3 calibration lines for 0.05 M H_2SO_4, 0.5 M Li_2SO_4, 0.1 M KOH and 1 M KOH, where 0.1 M KOH is performed in duplicates.

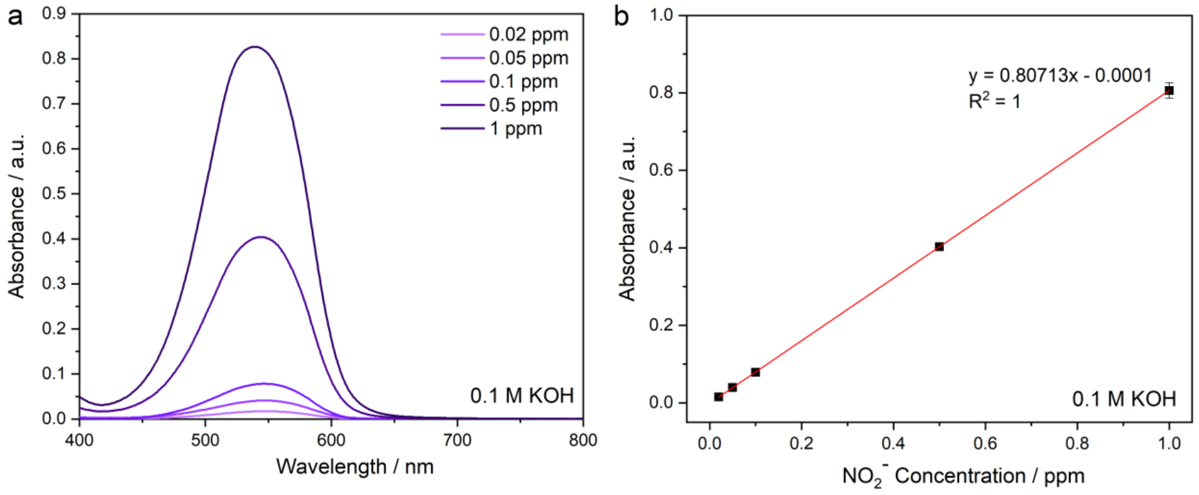

Figure S5. (a) UV-Vis spectra of 0 – 1 ppm NO_2^- concentrations in 0.1 M KOH with a maximum absorbance at 544 nm. (b) NO_2^- calibration line in 0.1 M KOH done in duplicates.

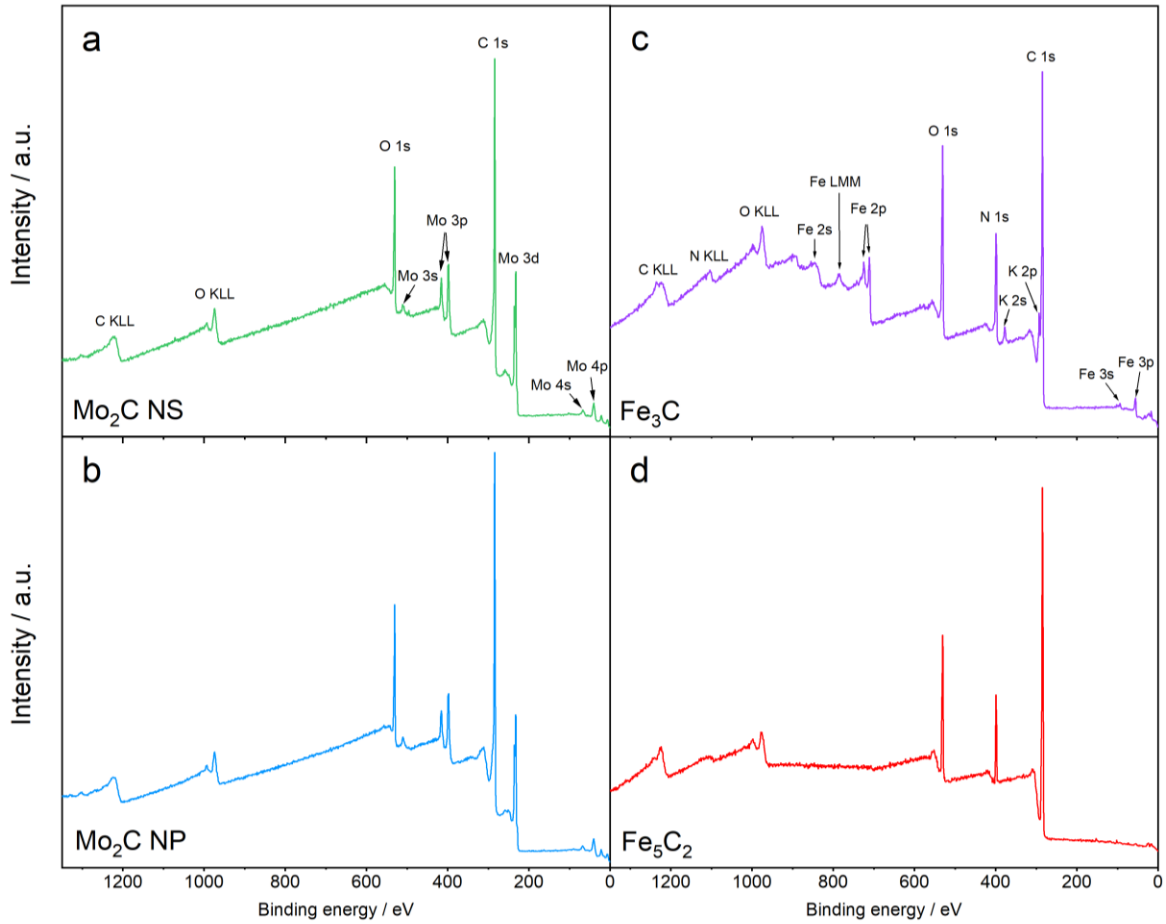

Figure S6. XPS survey of (a) Mo_2C NS, (b) Mo_2C NP, (c) Fe_3C and (d) Fe_5C_2 with peak allocation including auger peaks. The peaks were identified by the CasaXPS v2.3 database and ref [2].

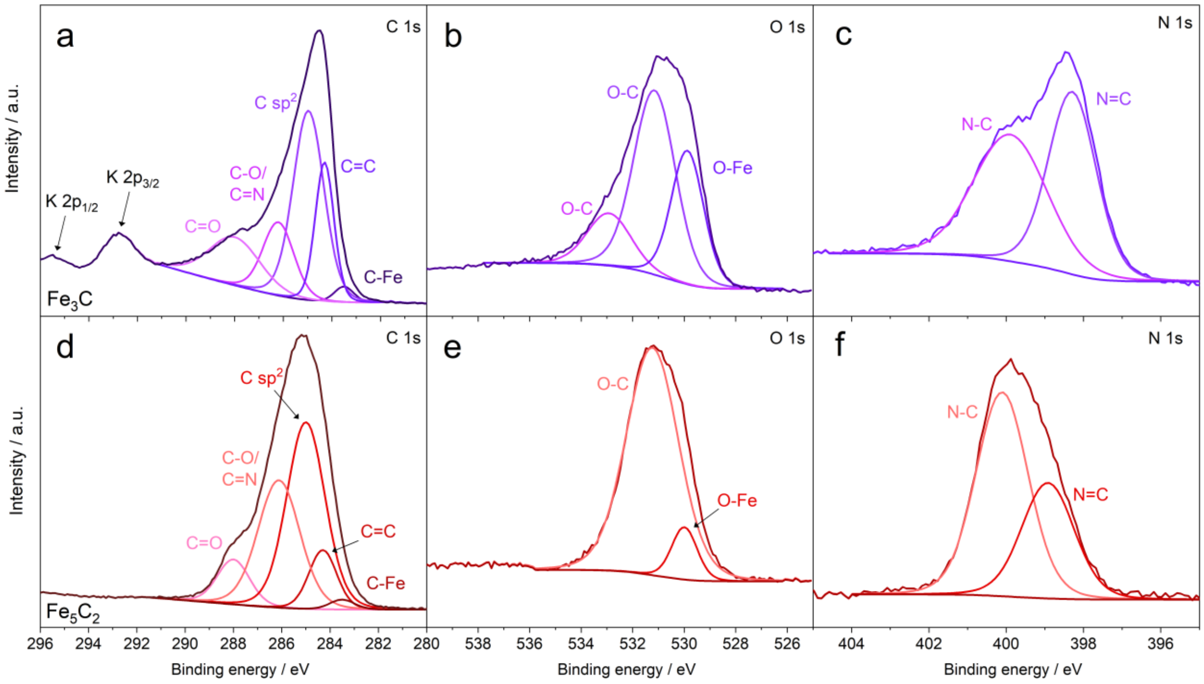

Figure S7. XPS spectra (a,d) C 1s, (b,e) O 1s, (c,f) N 1s of Fe_3C (purple) and Fe_5C_2 (red). Similar features as for the Mo_2C spectra, such as the absence of a clear carbide peak between 283-284 eV in the C 1s spectra and the identification of a Fe-oxide peak in the O 1s spectra due to air exposure. Two additional peaks in (a) at 292.8 eV and 295.5 eV were identified as K $2p_{1/2}$ and K $2p_{3/2}$, which are residual from the KOH wash.

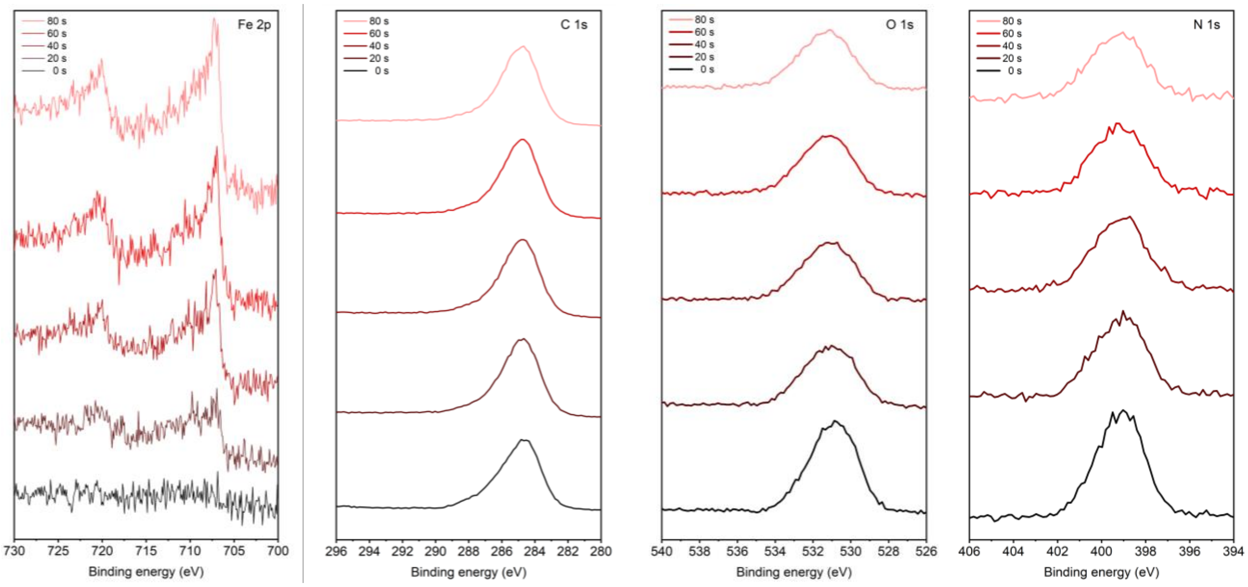

Figure S8. XPS depth profiling of Fe_5C_2 by in situ Ar^+ etching.

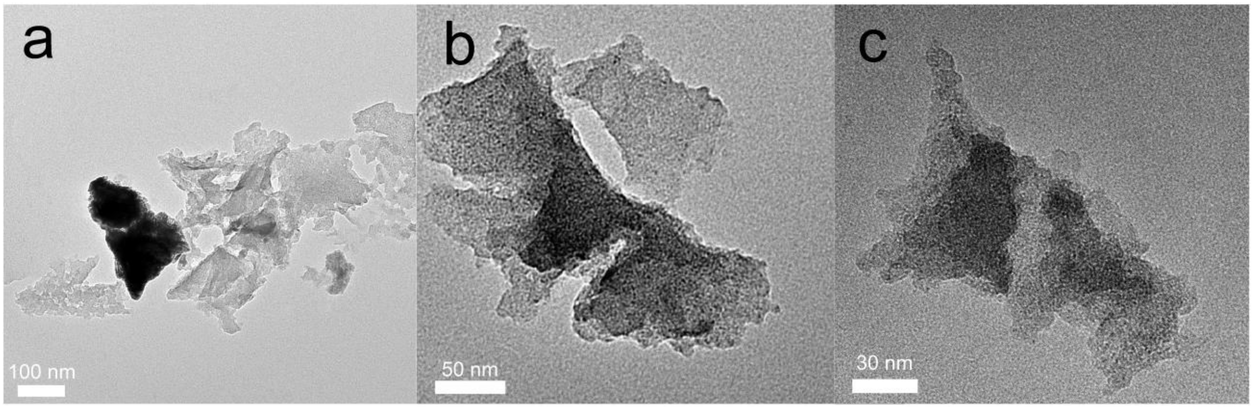

Figure S9. Transmission electron micrographs of various Mo_2C NS at different magnifications.

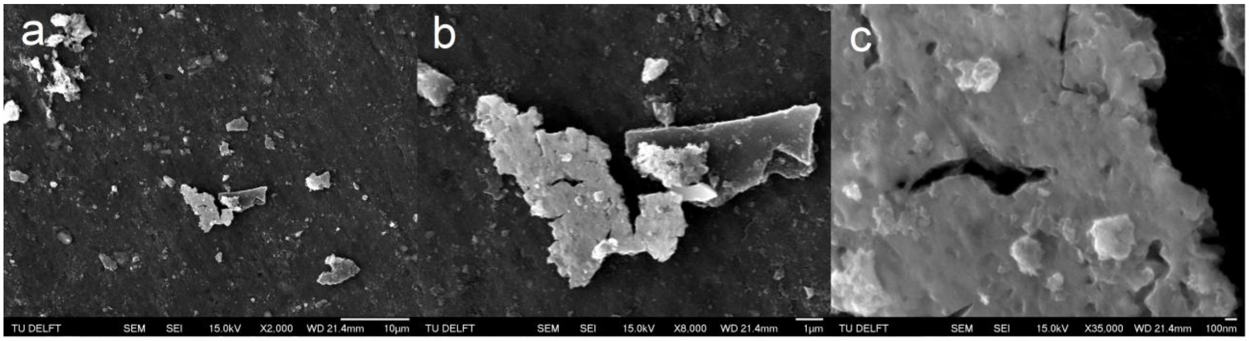

Figure S10. Scanning electron micrographs of one particular Mo_2C NS at different magnifications.

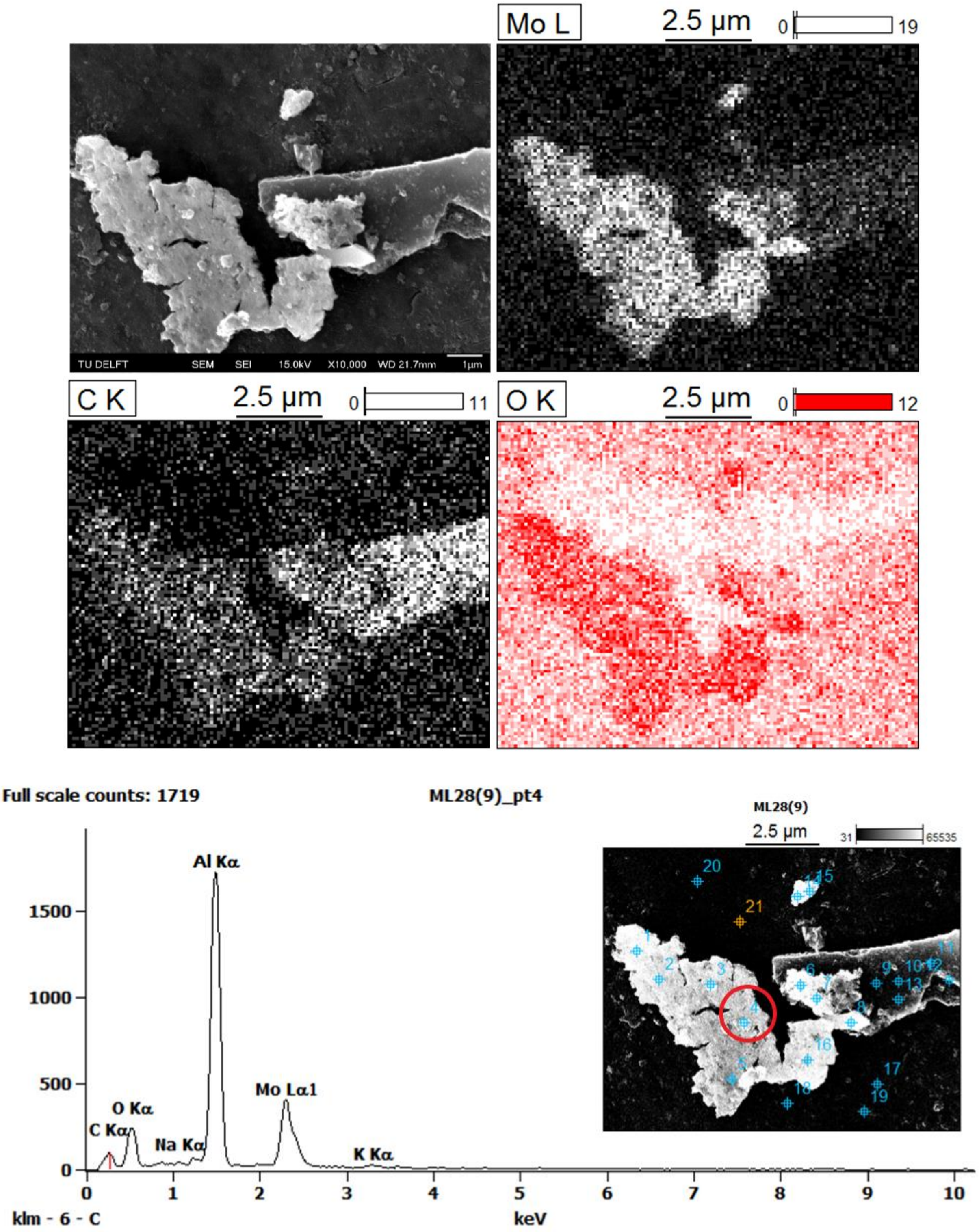

Figure S11. Energy-dispersive X-ray spectroscopy analysis of a Mo_2C NS. The spectra obtained by the point and shoot method at location 4 indicate a C, O and Mo peak at 0.28, 0.53 and 2.29 keV, respectively. The large peak at 1.49 keV is identified as the Al background signal from the supporting disc.

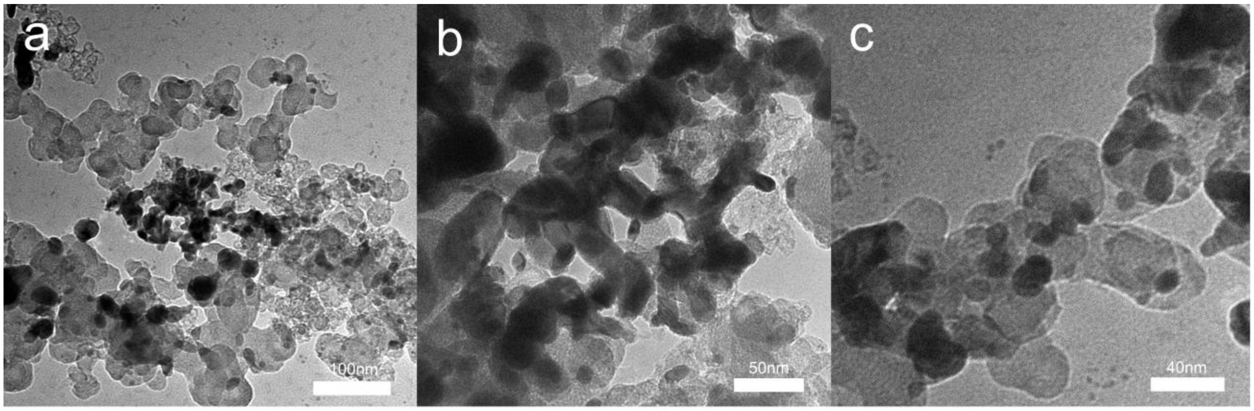

Figure S12. Transmission electron micrographs of Mo_2C NP anchored on a carbon support at different magnifications.

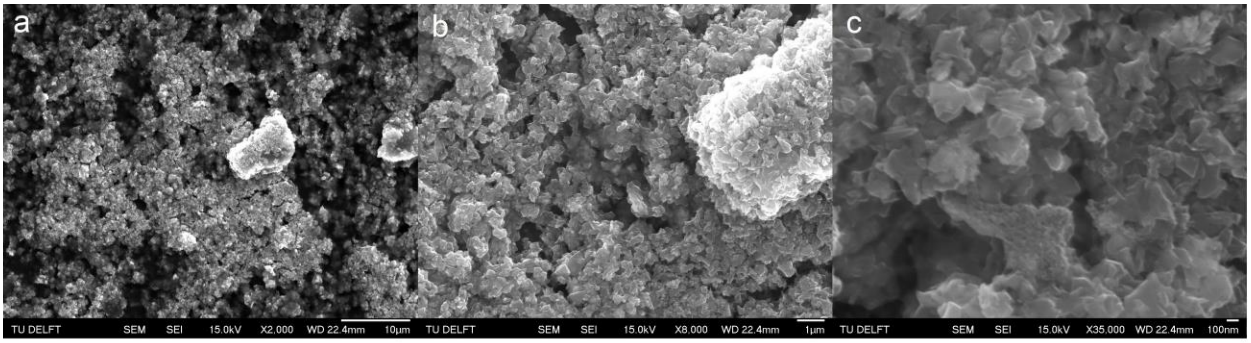

Figure S13. Scanning electron micrographs of the supported Mo_2C NP at different magnifications.

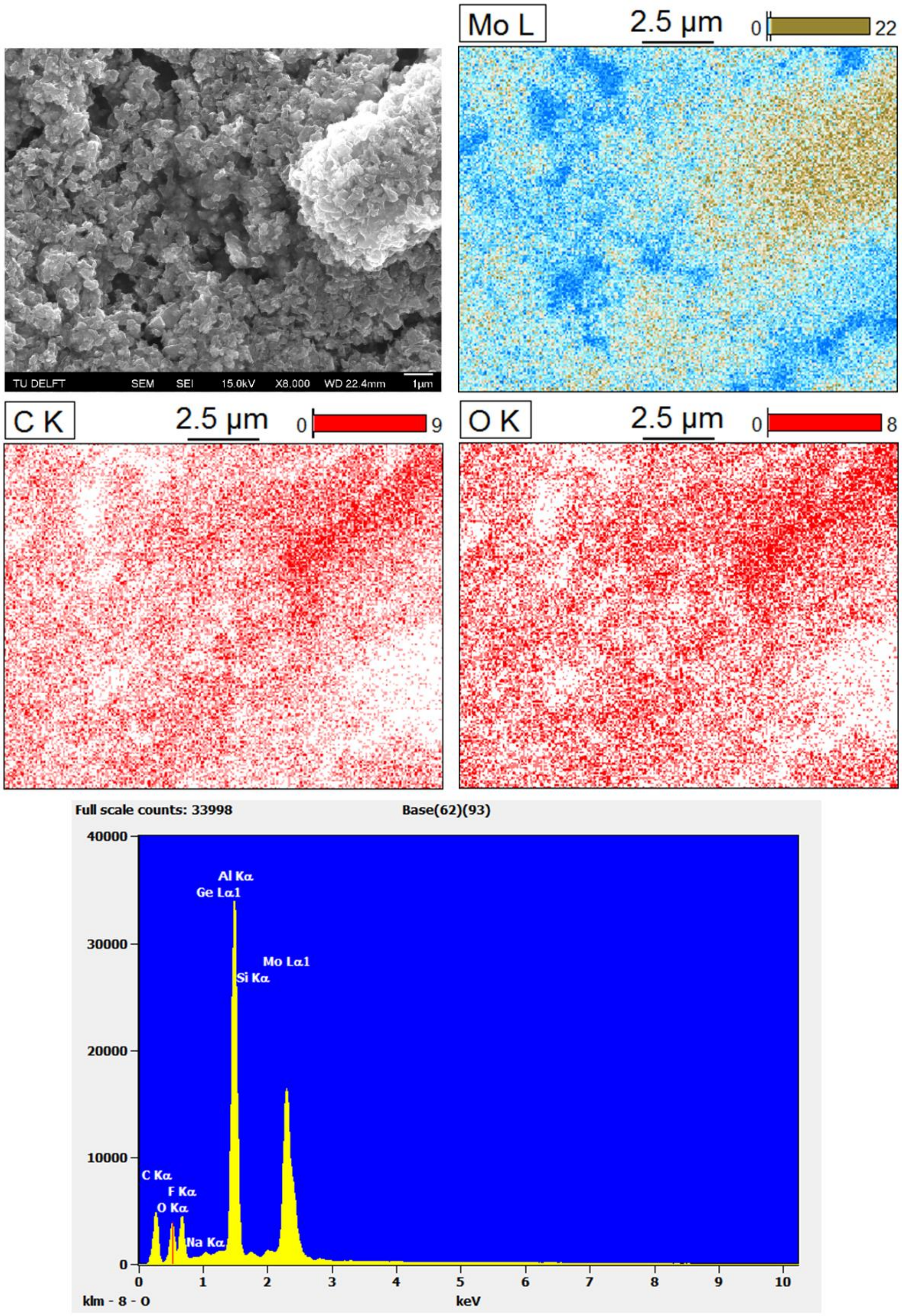

Figure S14. Energy-dispersive X-ray element mapping and spectra of Mo_2C NP. The EDX peaks at 0.28, 0.53 and 2.29 keV are assigned to C, O and Mo. The feature between 0.6-0.7 keV might be identified as F, but its origin remains unclear. The large peak at 1.49 keV is identified as the Al background signal from the supporting disc.

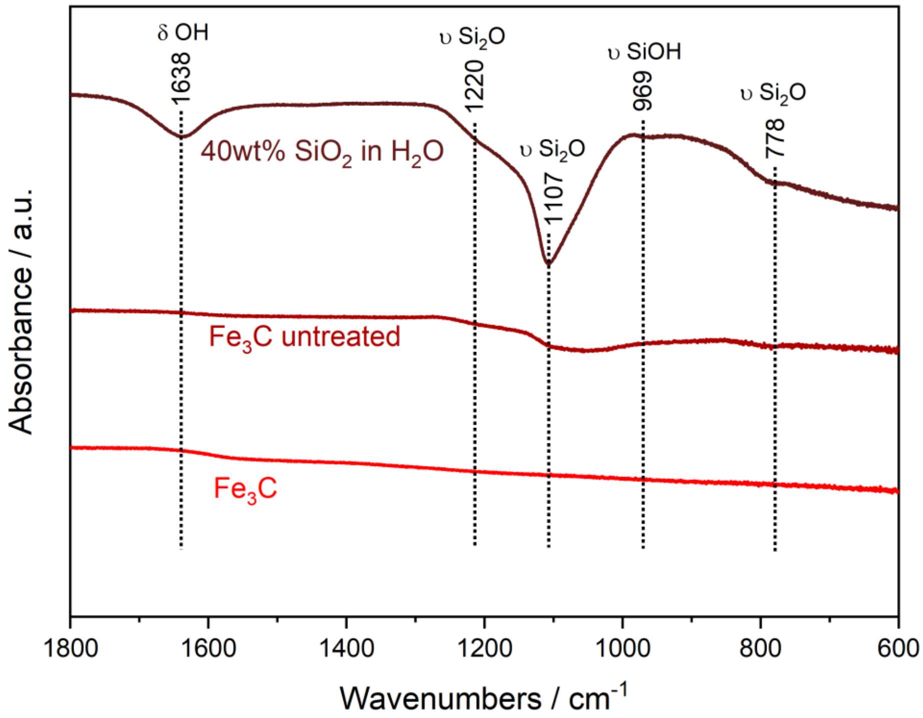

Figure S15. Attenuated total reflectance Fourier transform infrared (ATR-FTIR) spectroscopy was performed with a Thermo Scientific Nicolet iS50 FTIR spectrometer. The recorded spectra are 40wt% SiO_2 in H_2O (wine) as reference, Fe_3C without alkaline treatment (maroon) and Fe_3C treated with 1 M KOH (red). The shoulder feature at 1220 cm^{-1} and 1107 cm^{-1} from the reference spectra are both seen as asymmetric Si-O-Si stretching. Another small feature at 778 cm^{-1} is symmetric Si-O-Si stretching.[3] The spectra of the untreated Fe_3C is slightly bend in these regions and suggests the presence of the SiO_2 colloidal particles in the Fe_3C after the carburization procedure. The treated Fe_3C (red) did not show characteristic Si peaks, meaning that the Si phase was successfully removed.

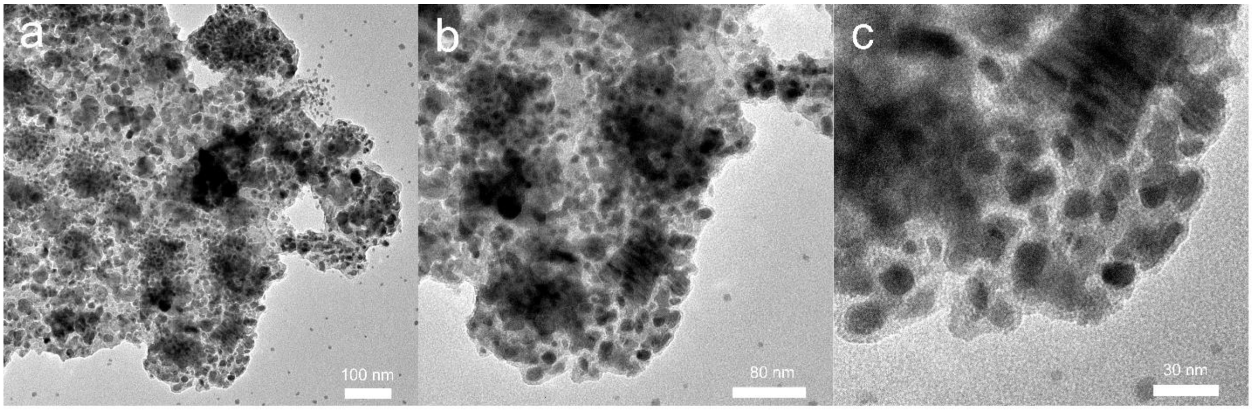

Figure S16. Transmission electron micrographs of Fe_5C_2 nanoparticles with increasing magnification from left to right.

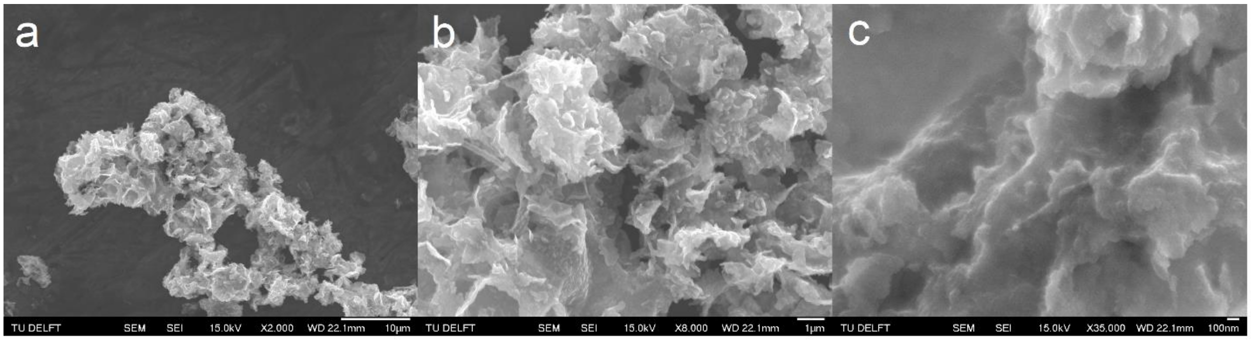

Figure S17. Scanning electron micrographs of Fe_5C_2 at different magnifications.

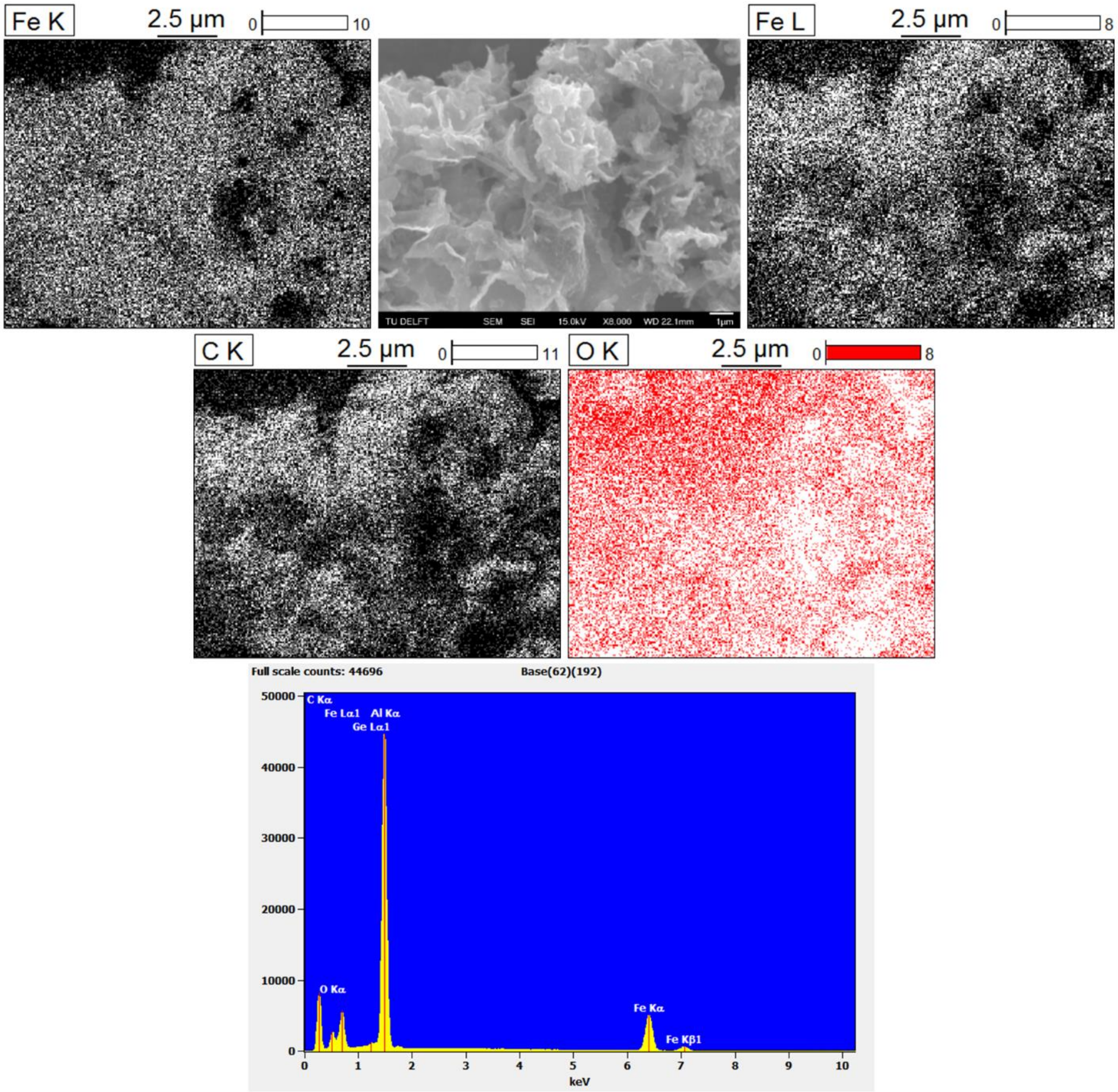

Figure S18. Energy-dispersive X-ray element mapping and spectra of Fe_5C_2. The spectra peaks between 0-1 keV are identified as C, O and Fe. Both the mapping and spectra show a sufficient distribution of Fe throughout the sample, which suggests that only the surface layer of materials contains a low quantity of Fe as observed by XPS. The large peak at 1.49 keV is identified as the Al background signal from the supporting disc.

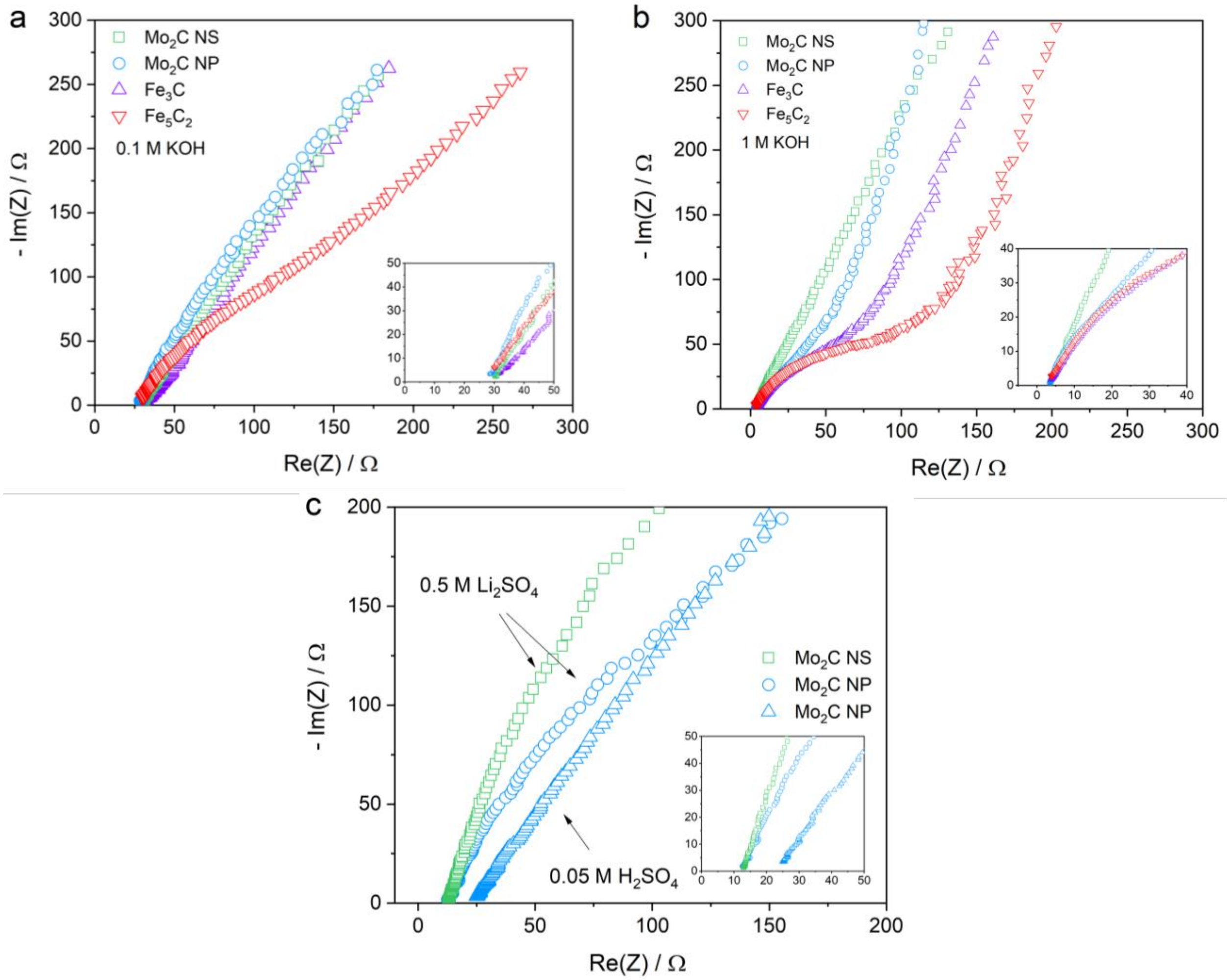

Figure S19. Nyquist plots at carried out at open-circuit conditions for different metal carbides and electrolytes, where R_u is estimated as the intersection with the Zreal axes. (a) 0.1 M KOH with R_u for Mo_2C NS = 30.1 Ω, Mo_2C NP = 28.4 Ω, Fe_3C = 30.8 Ω, Fe_5C_2 = 29.8 Ω; (b) 1 M KOH with R_u for Mo_2C NS = 3.6 Ω, Mo_2C NP = 3.4 Ω, Fe_3C = 3.6 Ω, Fe_5C_2 = 3.7 Ω; (c) 0.5 M Li_2SO_4 with R_u for Mo_2C NS = 12.8 Ω, Mo_2C NP = 12.6 Ω,; 0.05 M H_2SO_4 with R_u for Mo_2C NP = 25.0 Ω.

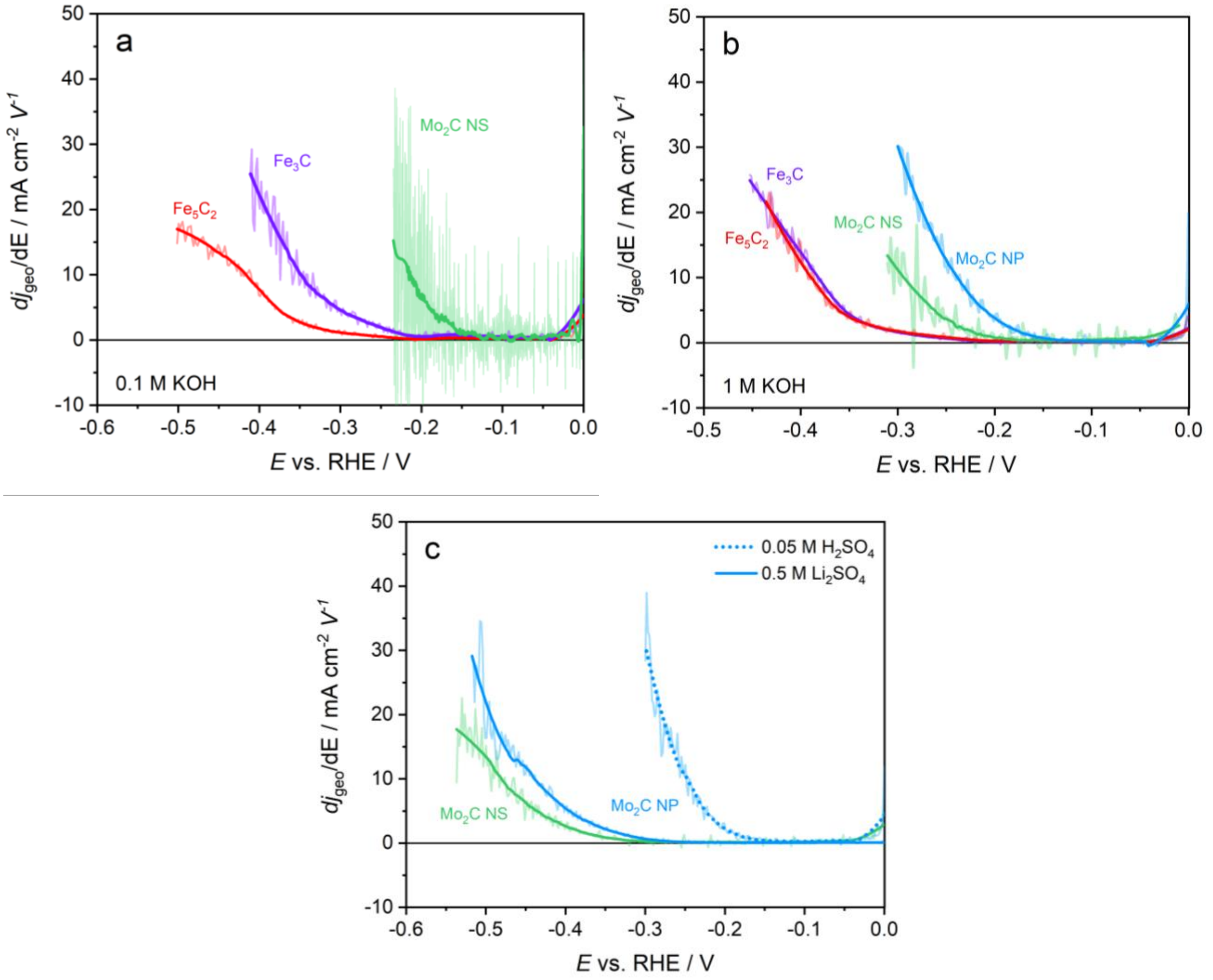

Figure S20. First derivative plot (dj/dE) vs. E of the cyclic voltammograms in **Figure 5**. The derivative was calculated with a build-in function in OriginPro. The derivative curves are plotted close to the onset potential (E_{onset}) region and show a significant level of noise, where we fitted an additional line for accurate determination. E_{onset} is defined in this work as the lift-off point of the fitted dj/dE curve from zero. (a) For 0.1M KOH, the derivative curve of Mo_2C NP is not plotted because the noise level was too high. The main issue was the very small time steps used during the recording of the CV, created a lot of data points that introduced a significant amount of noise. This was also observed for Mo_2C NS, but E_{onset} could still be estimated using data fitting. All E_{onset} below were converted to RHE scale. The following E_{onset} were obtained from the graph; Mo_2C NS = -0.13 V, Fe_3C = -0.22 V and Fe_5C_2 = -0.23 V. (b) For 1 M KOH; Mo_2C NS = -0.17 V, Mo_2C NP = -0.11 V, Fe_3C = -0.22 V and Fe_5C_2 = -0.22 V. (c) E_{onset} in 0.5 M Li_2SO_4 is Mo_2C NS = -0.32 V and Mo_2C NP = -0.28 V. E_{onset} for Mo_2C NP in 0.05 M H_2SO_4 is -0.17 V.

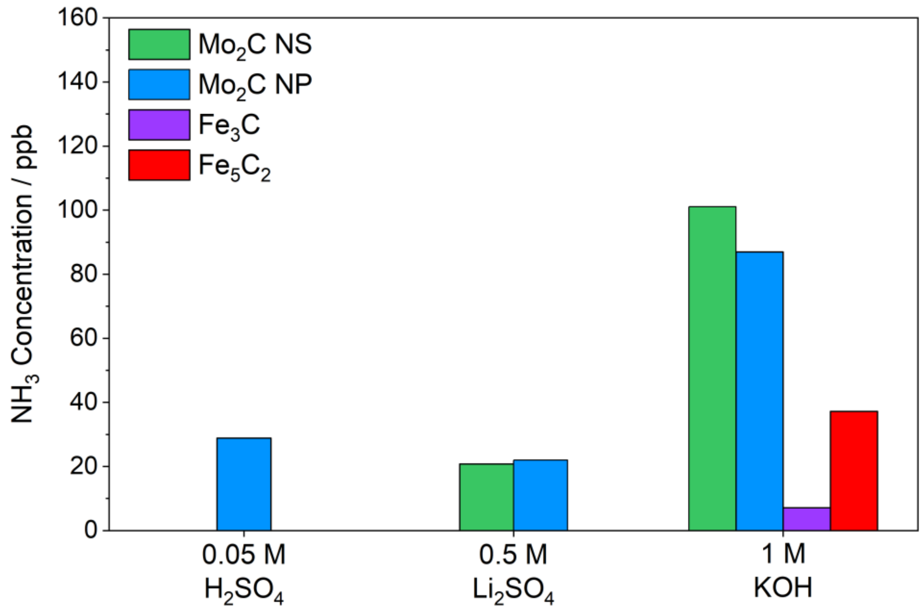

Figure S21. Qualitative analysis of the NRR activity of metal carbides at different pH. NH_3 concentrations were quantified after 40 cyclic voltammetry scans in a potential window where NRR is expected.

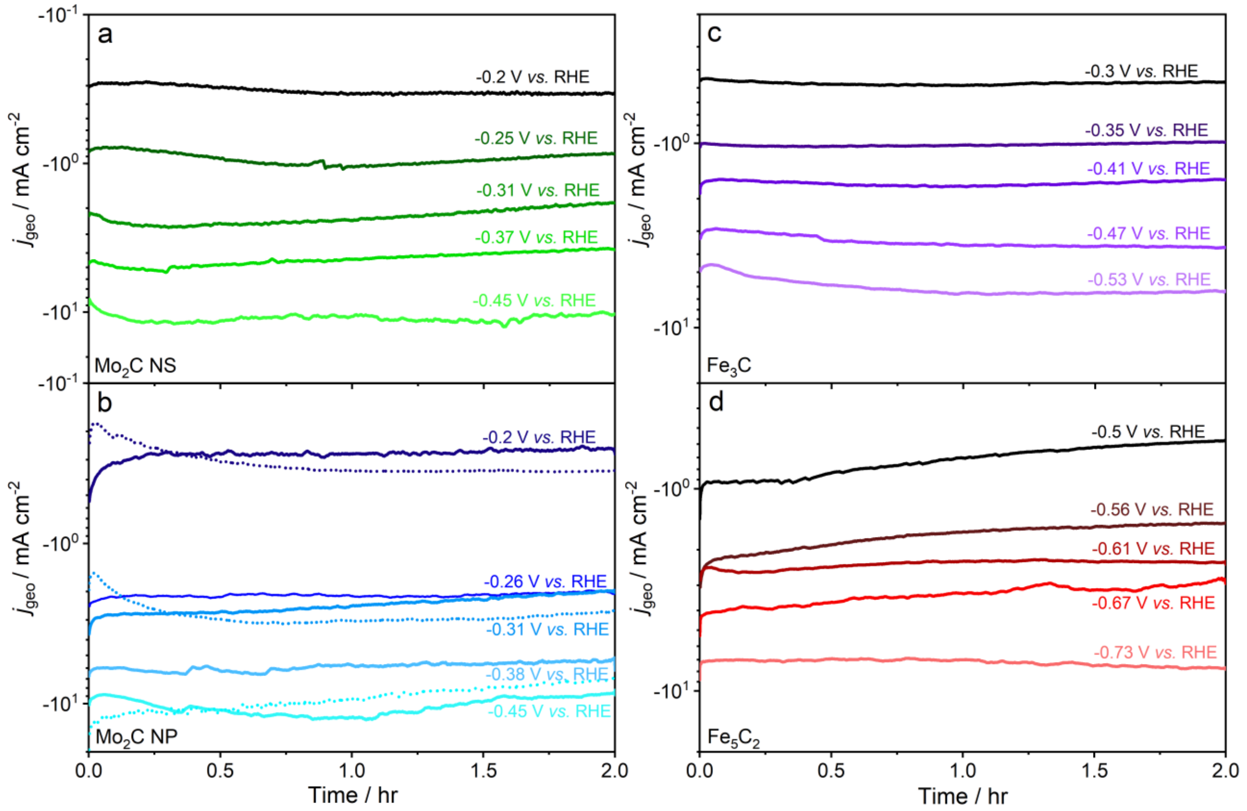

Figure S22. Chronoamperometry measurements at five different potentials (100% R_u compensated) in 0.1 M KOH with 0.06 mg·cm^{-2} catalyst loading of (a) Mo_2C NS, (b) Mo_2C NP, including three measurements with saturated Ar (dotted line), (c) Fe_3C and (d) Fe_5C_2 in 0.1 M KOH.

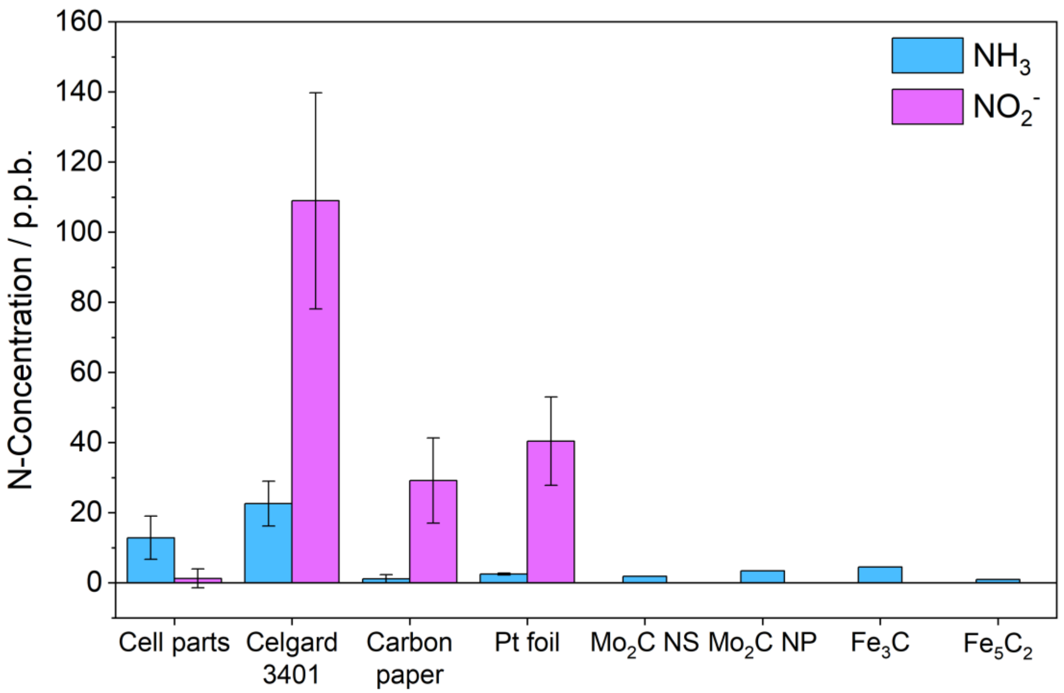

Figure S23. Quantification of several N-containing impurities after the cleaning procedure from several used cell components. After washing the PEEK cell body parts excessively with H_2O, the cell was assembled and filled with 10 ml 0.1 M KOH, then sealed off. The assembled cell was mounted for a duration of 15 min on a vortex shaker to trap the remaining impurities in the electrolyte. A 2.5 cm x 2.5 cm piece of Celgard 3401, 1.12 cm^2 carbon paper disk and 2.5x2.5cm^2 Pt foil were washed several times with H_2O and transferred to a separate test tube filled with 5 ml 0.1 M KOH. Subsequently, the test tubes were sonicated for 15 min. Afterwards, the impurities were directly quantified. The obtained results were extrapolated to the actual geometries of the used components in the electrochemical experiments in order to make a sound estimation of the level of background impurities after the cleaning procedure. Bar charts with error bars indicate the standard deviation of triplicates.

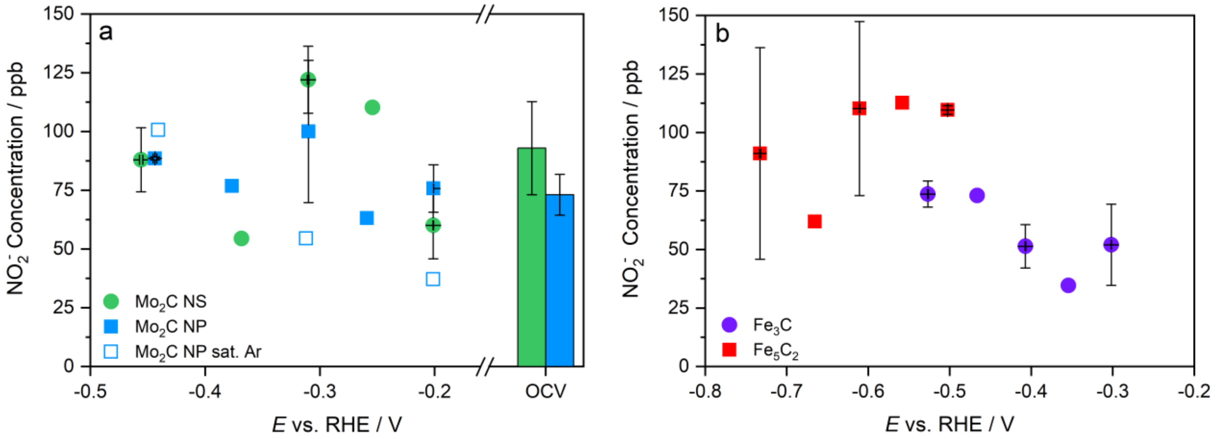

Figure S24. Quantified NO_2^- concentration after two hour chronoamperometry measurements in 0.1 M KOH corresponding to **Figure 6**. (a) Mo_2C NS (green, spherical) and Mo_2C NP in N_2 (blue, rectangular) and Ar (blue, open rectangular). (b) Fe_3C (purple, spherical) and Fe_5C_2 (red, rectangular). Data points with the error bars were done in duplicates.

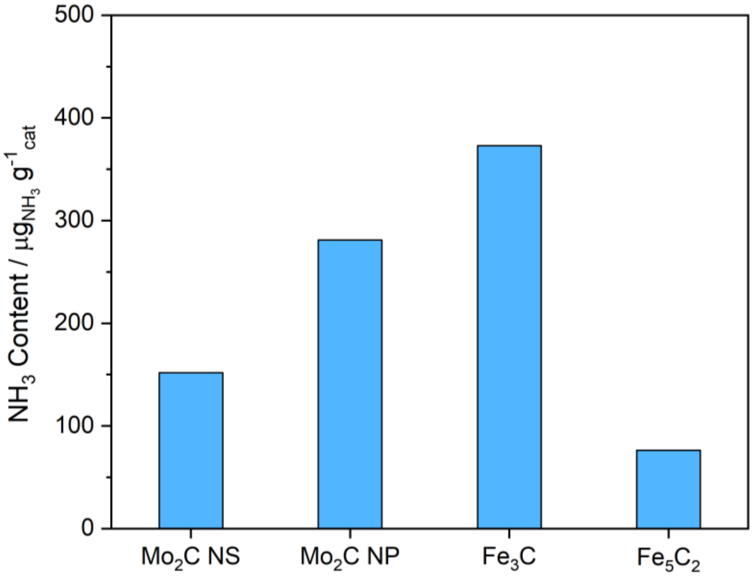

Figure S25. NH_3 impurities in the catalyst powders. For each material, 10 mg was dispersed in a sample tube filled with 5 ml 0.1 M KOH and sonicated for 15 min. Afterwards, the suspension was centrifuged for 15 min at 9000 rpm to separate the powder from the electrolyte in order to avoid major interference during the quantification process. Non-visible colloidal particles interfered most likely with the NO_2^- Griess test, therefore only NH_3 was quantified. An alternative method for the quantification of NO_2^- within various commercial metal powders has been implemented by Chen et al. but was not adopted in this work.[4]

Additional Supporting Figures

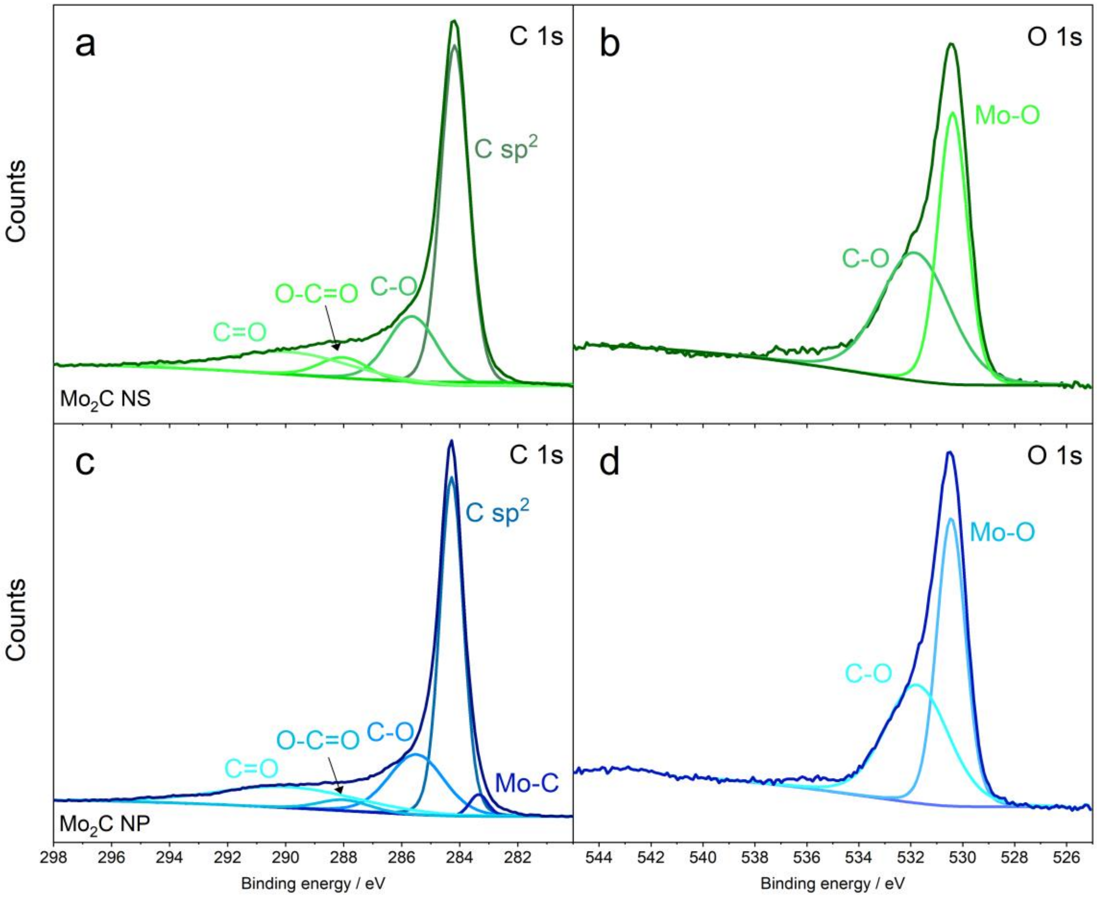

Figure S26. (a, c) C 1s and (b, d) O 1s spectra of Mo_2C NS (green) and Mo_2C NP (blue), respectively. The carbide phase (Mo-C) with a slightly lower binding energy as the C sp^2 bond could not be identified with great certainty. The role of adventitious hydrocarbon moieties might play a role due to exposure to air, which does also explain the relatively large Mo-oxide phase in the O 1s spectra. Another reason is the possible shielding effect by the excessive amount of carbon present in the support. The N 1s peak could not be deconvoluted because it overlaps with the Mo 3p peak.

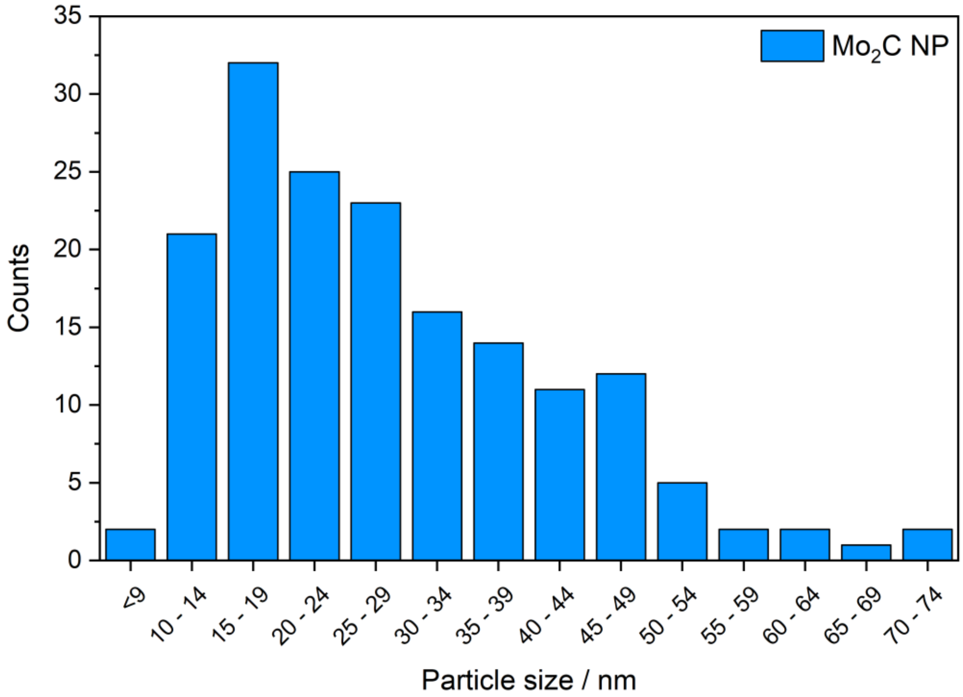

Figure S27. Mo_2C NP size distribution composed of 168 particles from four different TEM grid locations.

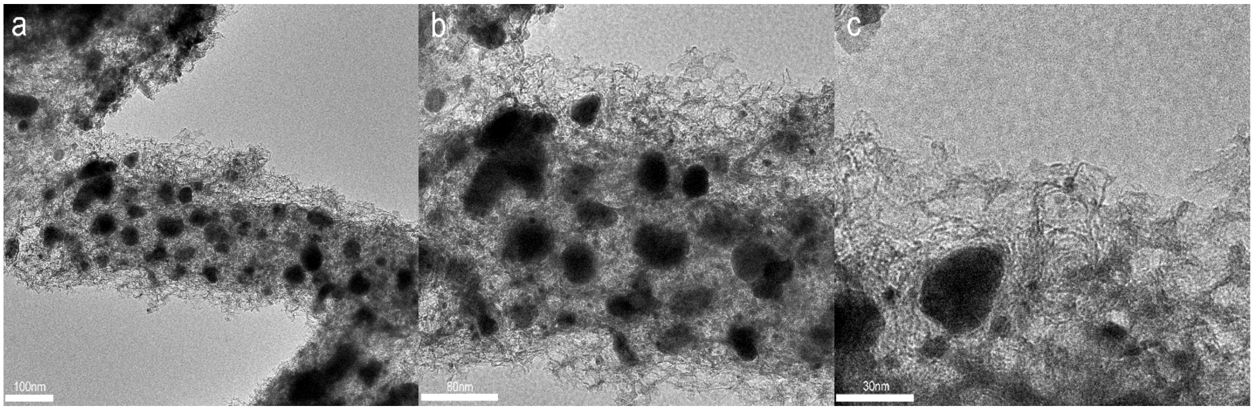

Figure S28. Transmission electron micrographs of the Fe_3C structure. The hollow features in the carbon support structure represent dissolved SiO_2 nanospheres (~20 nm) by alkaline treatment.

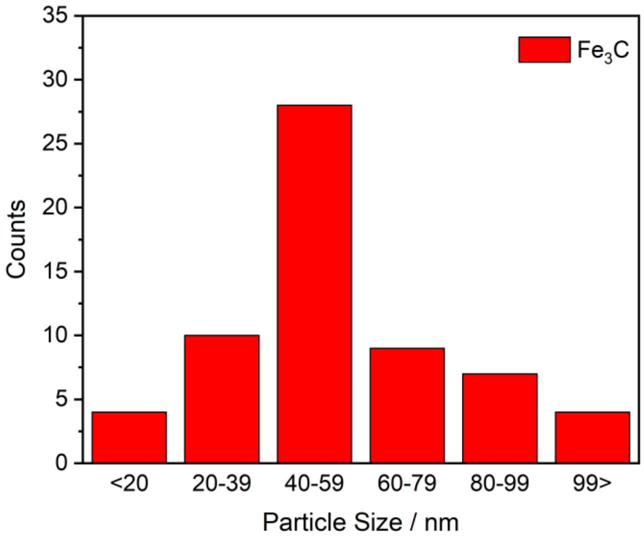

Figure S29. Fe_3C particle size distribution estimated by 62 particles at three different TEM grid locations.

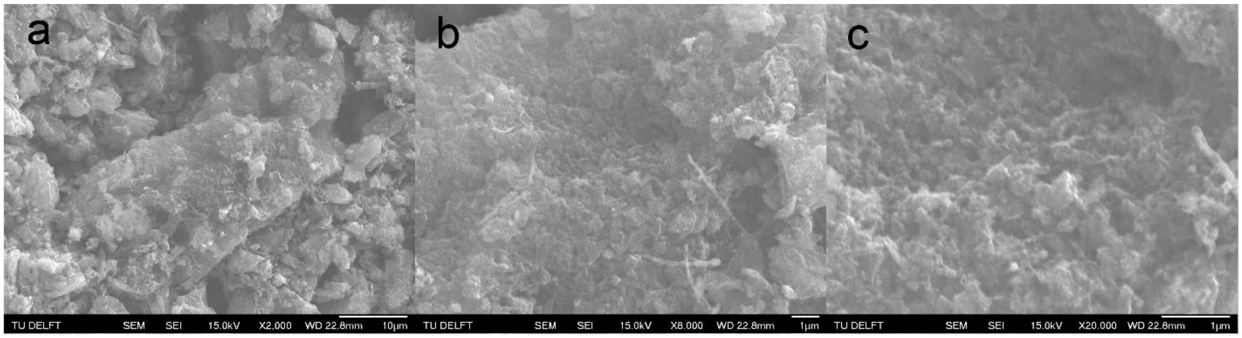

Figure S30. Scanning electron micrographs of the Fe_3C mesoporous surface structure, which agrees well with Giordano and coworkers.[5]

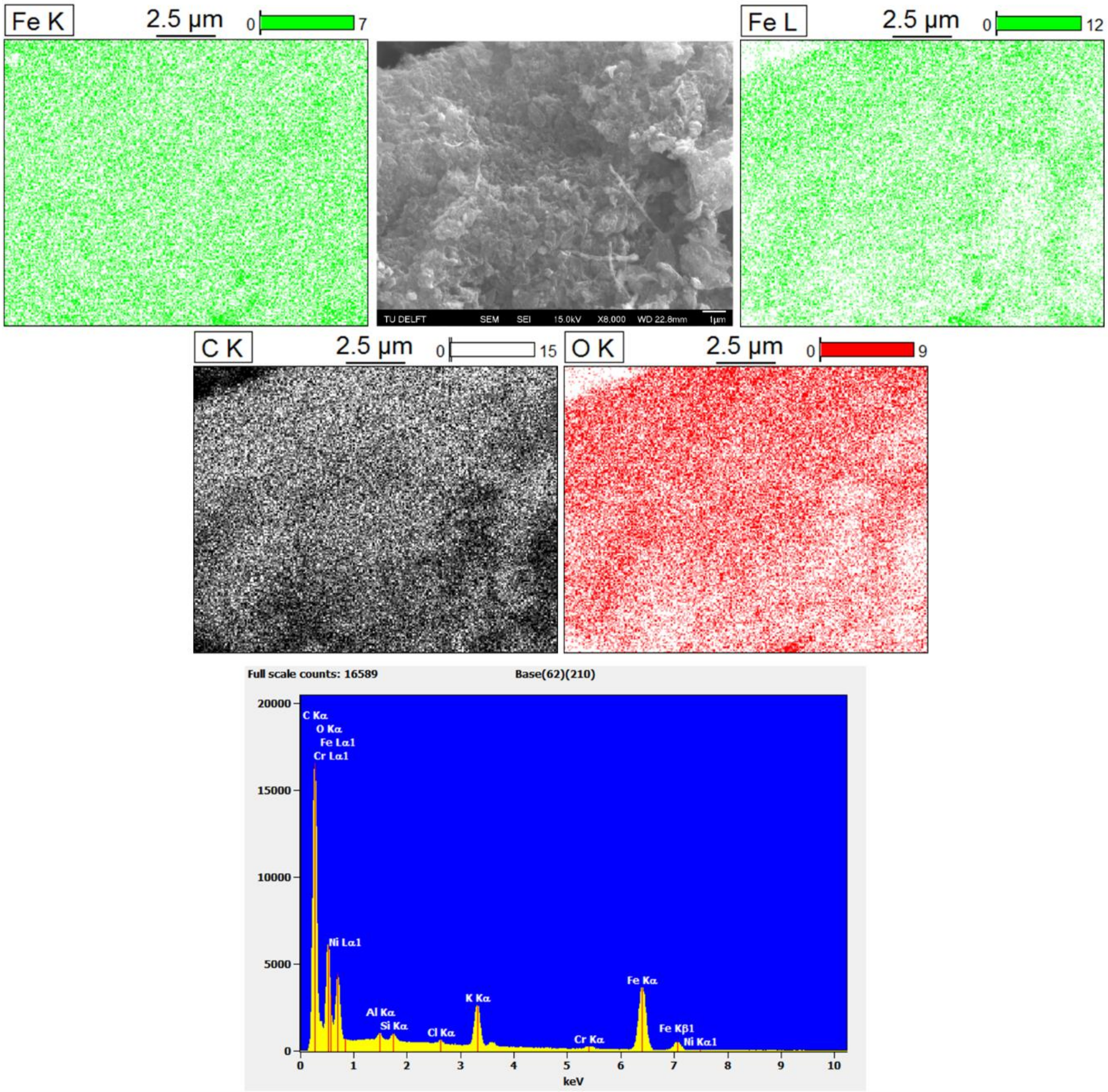

Figure S31. Energy-dispersive X-ray element mapping and spectra of the mesoporous Fe_3C. The EDX peaks at 0.28, 0.53 and 0.71 keV are assigned to C, O and Fe. Although small quantities of Cr and Ni were identified, it is unlikely that these species are present in the sample. The peak at 3.31 keV is the remaining K from the alkaline wash during the removal of SiO_2 particles, which appears to be effective due to the low intensity of the Si peak at 1.74 keV.

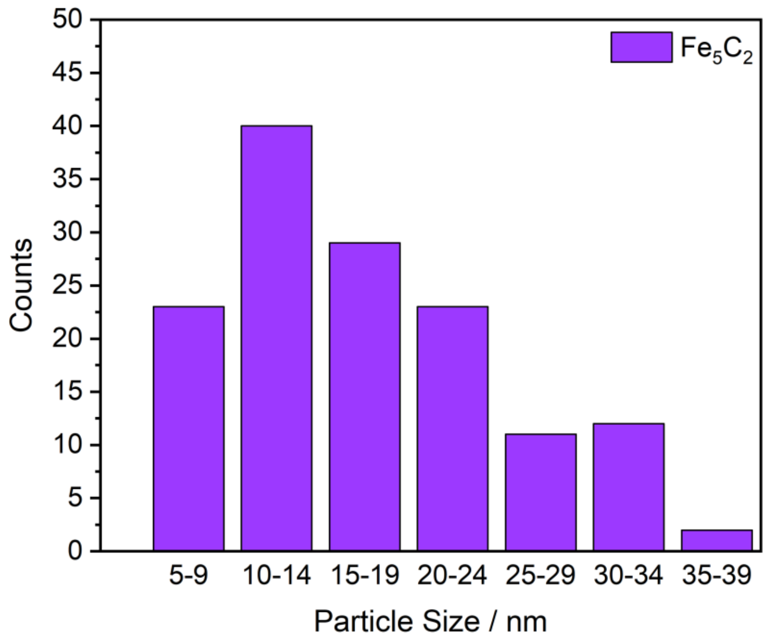

Figure S32. Fe_5C_2 particle size distribution of 161 particles collected from three different TEM grid locations. Particles lower than 5 nm could not be quantified due to the resolution limitations of the instrument.

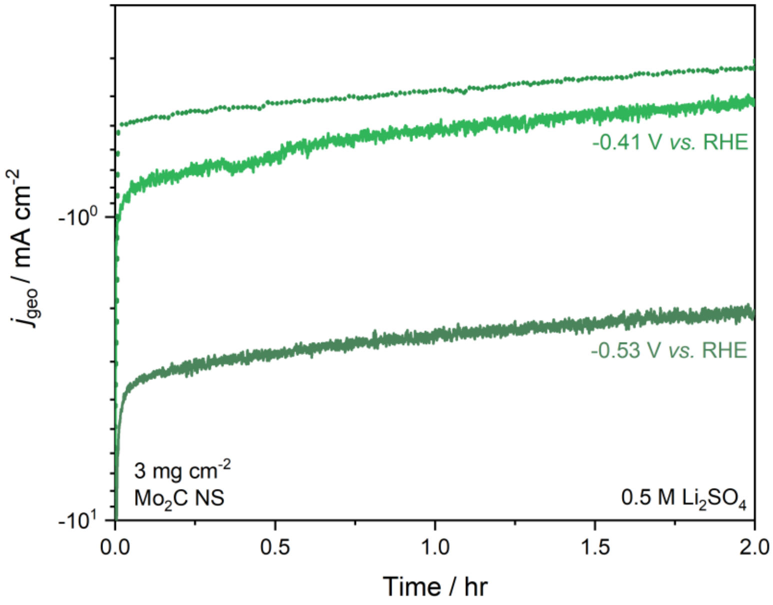

Figure S33. Chronoamperometry measurements of Mo_2C NS at -0.41 V and -0.53 V vs. RHE (100% R_u compensated) in 0.5 M Li_2SO_4 with 3 mg·cm^{-2} catalyst loading. The dotted line represents an Ar control experiment at -0.4 V vs. RHE.

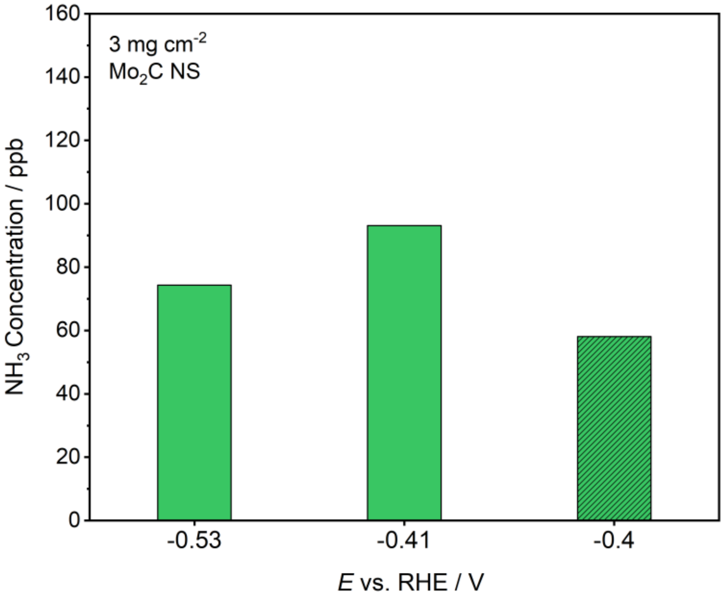

Figure S34. Quantified NH_3 concentrations after two hour chronoamperometry measurements with Mo_2C NS in 0.5 M Li_2SO_4 corresponding to **Figure S33**. The hatched histogram represents the NH_3 concentration after the Ar blank test.

Supporting Tables

Table S1. The average crystallite size estimated by the Scherrer equation.

	Peak (2θ)	FWHM (2θ)	$D_{Scherrer}$ (nm)
Mo_2C NS	40.35	0.16	35
	73.16	0.26	
	103.35	0.43	
Mo_2C NP	44.51	0.31	21
	61.49	0.37	
	83.05	0.45	
	98.20	0.54	
Fe_3C	52.92	0.18	42
	57.86	0.19	
	68.68	0.21	
	84.70	0.24	
Fe_5C_2	46.19	0.68	11
	52.70	0.78	
	67.43	0.88	
	69.16	0.88	

Table S2. The Mössbauer fitted parameters of the sample, obtained at 300 K. Experimental uncertainties: Isomer shift: I.S. ± 0.02 mm s^{-1}; Quadrupole splitting: Q.S. ± 0.02 mm s^{-1}; Line width: Γ ± 0.03 mm s^{-1}; Hyperfine field: ± 0.1 T; Spectral contribution: ± 3%.

Sample	IS ($mm \cdot s^{-1}$)	QS ($mm \cdot s^{-1}$)	Hyperfine field (T)	Γ ($mm \cdot s^{-1}$)	Phase	Spectral contribution (%)
Fe_3C	0.00	-	33.0	0.29	Fe^0	11
	0.19	-	20.8	0.38	θ-Fe_3C	67
	0.31	-0.01	49.3	0.63	γ-Fe_2O_3	7
	0.31	0.72	-	0.60	Fe^{3+}	15
Fe_5C_2	0.00	-	33.2	0.32	Fe^0	4
	0.27	-	21.7	0.51	χ-Fe_5C_2 (I)	32
	0.21	-	18.1	0.51	χ-Fe_5C_2 (II)	26
	0.16	-	10.3	0.51	χ-Fe_5C_2 (III)	20
	0.28	0.00	49.1	0.25	Fe_3O_4 (I)	2
	0.71	-0.07	46.0	0.37	Fe_3O_4 (II)	5
	0.27	0.88	-	0.64	Fe^{3+}	11

Table S3. Elemental Mo and Fe analysis by ICP-OES.

	Mo (wt%)	Fe (wt%)
Mo_2C NS	41.5	
Mo_2C NP	59.2	
Fe_3C		15.1
Fe_5C_2		12.9

Table S4. * calculated. None of the literature reports monitored NO_x or filtered the feed gasses. All sources used a Nafion membrane, except ref. [6] and [7].

Ref	Catalyst	Electrolyte	j_{NH_3} (mA cm^{-2})	E_w (V vs. RHE)	Yield rate (nmol s^{-1} cm^{-2})	(nmol s^{-1} mg^{-1}_{cat})	FE (%)	Loading (mg)	N^- in cat. or sup.	Dupl./ tripl.	Ar	OCV	$^{15}N_2$
8	Mo_3Fe_3C	0.1 M Li2SO4	-0.003	-0.05	0.016	0.020	27	0.8	Y	Y	Y	N	N
9	Mo_2C/C	0.5 M Li2SO4	-1.04	-0.3	0.930	0.093	7.8	3	N	N	N	N	Q
10	Fe_3Mo_3C/C	1 M KOH	-0.063	-0.5	0.214	0.021	0.05	3	N	N	Y	N	Q
11	Mo_2C/N-C	0.1 M Na2SO4	-0.002	-0.2	0.016	0.020	12.3	0.8	Y	Y	Y	N	N
12	Mo_2C film/Mo foil	0.1 M Na2SO4	-0.060	-0.55	0.055	-	40.2	-	Y	N	Y	Y	N
13	Mo/Mo_xC NP	0.1 M Na2SO4	-0.009	-0.3	0.166*	0.33	13	0.13	N	Y	Y	N	N
6	Fe-doped Mo_2C	0.5 M Na2SO4	-0.101	-0.45	0.006	0.61	20.1	0.01	Y	Y	Y	Y	N
14	Mo-Mo_2C@N-CNT	0.005 M H2SO4 + 0.1 M K2SO4	-0.047	-0.25	0.263	-	5.9	-	Y	N	N	N	Q
15	SA Mo@N-C	0.1 M KOH	-0.175	-0.3	0.213*	0.56	14.6	0.38	Y	Y	Y	N	Y
16	Mo_2C nanoflakes	Nafion	-0.011	-0.5	0.022	-	1.8	0.5	N	N	Y	Y	N
17	O-MoC@N-C	0.1 mM HCl + 0.5 M LiSO4	-0.038	-0.35	0.13	0.37	25.1	0.14	Y	Y	Y	Y	N
18	Fe_3C/C	0.05 H2SO4	-0.092	-0.2	0.209	0.14	9.15	0.15	N	N	Y	Y	N
19	Fe_3C NP@N-C	0.1 M KOH	-0.064	-0.4	0.098	0.26	2.72	0.38	Y	Y	Y	Y	Q
7	$Fe_3C/Fe_2O_3/Fe/C$	6 M KOH	0.004	0.1	0.0049	0.005	0.38	1	Y	Y	Y	Y	Y
	Mo_2C NS	0.1 M KOH	-0.001	-0.2	0.004	0.065	0.41	0.06	N	Y	Y	Y	N
	(This work)	0.5 M Li_2SO_4	-0.001	-0.41	0.003	0.001	0.18	3	N	N	Y	N	N
	Mo_2C NP (This work)	0.1 M KOH	-0.001	-0.2	0.004	0.065	0.42	0.06	Y	Y	N	Y	N
	Fe_3C/Fe-oxide/Fe (This work)	0.1 M KOH	-0.0005	-0.3	0.0017	0.028	0.11	0.06	Y	Y	N	N	N
	Fe_5C_2/Fe-oxide/Fe (This work)	0.1 M KOH	-0.0005	-0.5	0.0016	0.026	0.077	0.06	Y	Y	N	N	N

Table S5. XPS peak deconvolution details of molybdenum carbide. The line shape of the curves were approximated as a Lorentzian with LA(1.53,243) with a U2 Tougaard background type.

Material	Orbital	Peak	Binding Energy (eV)	FWHM (eV)	Relative area (%)	at %	wt %
Mo_2C NS	Mo 3d	$Mo^{0-3+}{}_{5/2}$	229.29	1.29	5.87	5.6	31.0
		$Mo^{0-3+}{}_{3/2}$	232.44	1.29	3.91		
		$Mo^{4+}{}_{5/2}$	230.88	1	3.36		
		$Mo^{4+}{}_{3/2}$	234.03	1	2.24		
		$Mo^{6+}{}_{5/2}$	232.45	1.56	48.31		
		$Mo^{6+}{}_{3/2}$	235.60	1.56	32.21		
	C 1s	C sp^2	284.17	1.06	46.56	78.6	54.4
		C-O	285.12	2.83	32.16		
		O-C=O	288	2.41	6.15		
		C=O	290	5.00	15.42		
	O 1s	O-Mo	530.39	1.32	26.40	15.8	14.6
		O-C	531.84	3.15	63.00		
Mo2C NP	Mo 3d	$Mo^{0-3+}{}_{5/2}$	228.22	1.01	4.13	4.8	27.9
		$Mo^{0-3+}{}_{3/2}$	231.37	1.01	2.75		
		$Mo^{4+}{}_{5/2}$	229.40	0.94	2.46		
		$Mo^{4+}{}_{3/2}$	232.55	0.94	1.64		
		$Mo^{6+}{}_{5/2}$	232.44	2.03	52.15		
		$Mo^{6+}{}_{3/2}$	235.57	2.03	34.77		
	C 1s	C-Mo	283.35	0.74	2.67	83.2	60.5
		C sp^2	284.28	0.96	52.49		
		C-O	285.5	2.25	21.94		
		O-C=O	288	2.05	3.66		
		C=O	289.95	5.75	19.43		
	O 1s	O-Mo	530.45	1.30	51.82	12.0	11.6
		O-C	531.77	2.88	47.20		

Table S6. XPS peak deconvolution details of iron carbide. The line shape of the curves were approximated as a Lorentzian with LA(1.53,243) with a Shirley background type.

Material	Orbital	Peak	Binding Energy (eV)	FWHM (eV)	Relative area (%)	at%	wt%
Fe_3C	Fe 2p	Fe_3C	708.37	2.00	6.75	2.9	11.4
		$Fe_2O_3{}^1$	709.80	1.20	26.63		
		$Fe_2O_3{}^2$	710.75	1.20	24.50		
		$Fe_2O_3{}^3$	711.67	1.31	18.77		
		$Fe_2O_3{}^4$	712.65	1.40	10.04		
		$Fe_2O_3{}^5$	713.71	2.20	9.93		
	C 1s	Fe-C	283.50	0.93	2.27	68.6	58.2
		C sp^2	284.30	0.91	19.42		
		C sp^3	284.96	1.50	43.63		
		C-O/C-N	286.20	1.50	16.89		
		C=O	288.00	2.47	18.24		
	O 1s	Fe-O	529.88	1.49	30.52	16	18.0
		C-O	531.15	1.91	54.02		
		C-O	532.92	2.00	16.11		
	N 1s	C-N-6 / $-NH_3$	398.29	1.46	48.96	12.6	12.4
		C-N-5 / $-NH_2$	399.89	2.24	52.04		
Fe_5C_2	Fe 2p	Fe^0	707.24	1.30	28.41	0.3	1.2
		Fe_3C	708.61	0.97	9.63		
		$Fe_2O_3{}^1$	709.80	1.20	17.11		
		$Fe_2O_3{}^2$	710.73	1.20	15.74		
		$Fe_2O_3{}^3$	711.78	1.40	12.06		
		$Fe_2O_3{}^4$	712.89	1.40	6.45		
		$Fe_2O_3{}^5$	713.97	2.29	6.38		
	C 1s	C-Fe	283.50	1.22	1.56	77.6	72.7
		C sp^2	284.30	1.36	10.42		
		C sp^3	285.00	1.87	45.24		
		C-O/C=N/C-OH	286.12	2.04	33.74		
		C=O	288.00	1.58	9.18		
	O 1s	Fe-O	529.99	1.17	10.14	12.2	15.2
		C-O	531.23	2.39	90.38		
	N 1s	C-N-6/ $-NH_3$	398.92	1.60	35.40	10.0	10.9
		C-N-5/ $-NH_2$	400.10	1.59	62.15		

Supporting Information References

(1) Kuhl, K. P.; Cave, E. R.; Abram, D. N.; Jaramillo, T. F. New insights into the electrochemical reduction of carbon dioxide on metallic copper surfaces. *Energy & Environmental Science* **2012**, *5* (5), 7050-7059.
(2) Chastain, J.; King Jr, R. C. Handbook of X-ray photoelectron spectroscopy. *Perkin-Elmer Corporation* **1992**, *40*, 221.
(3) Mayerhöfer, T. G.; Shen, Z.; Leonova, E.; Edén, M.; Kriltz, A.; Popp, J. Consolidated silica glass from nanoparticles. *Journal of Solid State Chemistry* **2008**, *181* (9), 2442-2447.
(4) Chen, Y.; Liu, H.; Ha, N.; Licht, S.; Gu, S.; Li, W. Revealing nitrogen-containing species in commercial catalysts used for ammonia electrosynthesis. *Nature Catalysis* **2020**, 1-7.
(5) Kraupner, A.; Markus, A.; Palkovits, R.; Schlicht, K.; Giordano, C. Mesoporous Fe3C sponges as magnetic supports and as heterogeneous catalyst. *Journal of Materials Chemistry* **2010**, *20* (29), 6019-6022.
(6) Wang, T.; Kou, Z.; Zhang, J.; Wang, H.; Zeng, Y. J.; Wei, S.; Zhang, H. Boosting Faradic efficiency of dinitrogen reduction on the negatively charged Mo sites modulated via interstitial Fe doping into a Mo2C nanowall catalyst. *Chemical Engineering Journal* **2021**, *417*, 127924-127924.
(7) Kim, J. H.; Ju, H.; An, B.-S.; An, Y.; Cho, K.; Kim, S. H.; Bae, Y.-S.; Yoon, H. C. Comparison between Fe2O3/C and Fe3C/Fe2O3/Fe/C Electrocatalysts for N2 Reduction in an Alkaline Electrolyte. *ACS Applied Materials & Interfaces* **2021**, acsami.1c20807-acsami.20801c20807.
(8) Qin, B.; Li, Y.; Zhang, Q.; Yang, G.; Liang, H.; Peng, F. Understanding of nitrogen fixation electro catalyzed by molybdenum–iron carbide through the experiment and theory. *Nano Energy* **2020**, *68*, 104374.
(9) Cheng, H.; Ding, L. X.; Chen, G. F.; Zhang, L.; Xue, J.; Wang, H. Molybdenum Carbide Nanodots Enable Efficient Electrocatalytic Nitrogen Fixation under Ambient Conditions. *Advanced Materials* **2018**, *30* (46), 1-7.
(10) Cheng, H.; Cui, P.; Wang, F.; Ding, L. X.; Wang, H. High Efficiency Electrochemical Nitrogen Fixation Achieved with a Lower Pressure Reaction System by Changing the Chemical Equilibrium. *Angewandte Chemie - International Edition* **2019**, *58* (43), 15541-15547.
(11) Zhang, Y.; Hu, J.; Zhang, C.; Cheung, A. T. F.; Zhang, Y.; Liu, L.; Leung, M. K. H. Mo2C embedded on nitrogen-doped carbon toward electrocatalytic nitrogen reduction to ammonia under ambient conditions. *International Journal of Hydrogen Energy* **2021**, *46* (24), 13011-13019.
(12) Ba, K.; Wang, G.; Ye, T.; Wang, X.; Sun, Y.; Liu, H.; Hu, A.; Li, Z.; Sun, Z. Single Faceted Two-Dimensional Mo 2 C Electrocatalyst for Highly Efficient Nitrogen Fixation. **2020**.
(13) Liu, Y.; Zhu, X.; Zhang, Q.; Tang, T.; Zhang, Y.; Gu, L.; Li, Y.; Bao, J.; Dai, Z.; Hu, J. S. Engineering Mo/Mo2C/MoC hetero-interfaces for enhanced electrocatalytic nitrogen reduction. *Journal of Materials Chemistry A* **2020**, *8* (18), 8920-8926.
(14) Ma, Y.; Yang, T.; Zou, H.; Zang, W.; Kou, Z.; Mao, L.; Feng, Y.; Shen, L.; Pennycook, S. J.; Duan, L.; et al. Synergizing Mo Single Atoms and Mo2C Nanoparticles on CNTs Synchronizes Selectivity and Activity of Electrocatalytic N2 Reduction to Ammonia. *Advanced Materials* **2020**, *32* (33), 1-8.

(15) Han, L.; Liu, X.; Chen, J.; Lin, R.; Liu, H.; Fang, L. U.; Bak, S.; Liang, Z.; Zhao, S.; Stavitski, E.; et al. Atomically Dispersed Molybdenum Catalysts for Efficient Ambient Nitrogen Fixation. *Angewandte Chemie - International Edition* **2019**, *58* (8), 2321-2325.

(16) Ramaiyan, K. P.; Ozden, S.; Maurya, S.; Kelly, D.; Babu, S. K.; Benavidez, A.; Garzon, F. G.; Kim, Y. S.; Kreller, C. R.; Mukundan, R. Molybdenum Carbide Electrocatalysts for Electrochemical Synthesis of Ammonia from Nitrogen: Activity and Stability. *Journal of The Electrochemical Society* **2020**, *167* (4), 044506-044506.

(17) Qu, X.; Shen, L.; Mao, Y.; Lin, J.; Li, Y.; Li, G.; Zhang, Y.; Jiang, Y.; Sun, S. Facile Preparation of Carbon Shells-Coated O-Doped Molybdenum Carbide Nanoparticles as High Selective Electrocatalysts for Nitrogen Reduction Reaction under Ambient Conditions. *ACS Applied Materials & Interfaces* **2019**, *11* (35), 31869-31877.

(18) Peng, M.; Qiao, Y.; Luo, M.; Wang, M.; Chu, S.; Zhao, Y.; Liu, P.; Liu, J.; Tan, Y. Bioinspired Fe3C@C as Highly Efficient Electrocatalyst for Nitrogen Reduction Reaction under Ambient Conditions. *ACS Applied Materials and Interfaces* **2019**, *11* (43), 40062-40068.

(19) Cong, L.; Yu, Z.; Liu, F.; Huang, W. Electrochemical synthesis of ammonia from N 2 and H 2 O using a typical non-noble metal carbon-based catalyst under ambient conditions †. *Cite this: Catal. Sci. Technol* **2019**, *9*, 1208-1208.
